# Identification of novel DNA gyrase B inhibitors in *Mycobacterium species* using integrated computational approaches

**DOI:** 10.3389/ftubr.2026.1854329

**Published:** 2026-06-18

**Authors:** Jagritee Yadav, Lalhmangaihzuali, Harish Shukla, Anshuman Chandra, Sudhir Kumar Singh, Meenakshi Singh

**Affiliations:** 1 Department of Medicinal Chemistry, Faculty of Ayurveda, Institute of Medical Sciences, Banaras Hindu University, Varanasi, India; 2 Department of Biochemistry, North Eastern Hill University, Shillong, Meghalaya, India; 3 School of Physical Sciences, Jawaharlal Nehru University, New Delhi, India; 4 Department of Microbiology, Virus Research and Diagnostic Laboratory (VRDL), Institute of Medical Sciences, Banaras Hindu University, Varanasi, India

**Keywords:** anti-tubercular, density functional theory (DFT), DNA Gyrase B, molecular docking, molecular dynamics, pharmacophore modeling, virtual screening

## Abstract

**Introduction:**

Drug-resistant Mycobacterium tuberculosis remains a major global health challenge, particularly due to the emergence of multidrug-resistant and extensively drug-resistant strains. DNA gyrase, specifically the ATPase domain of GyrB, represents a promising target for the development of novel anti-tubercular agents.

**Methods:**

A comprehensive computational workflow was employed, including pharmacophore-based virtual screening of approximately 1,000,000 compounds from the PubChem, ZINC, ChEMBL, and MolPort databases. Hits were filtered using Lipinski's rule of five, structural diversity analysis, molecular docking, ADMET and toxicity assessment, molecular dynamics simulations, and density functional theory analyses.

**Results:**

Approximately 30,000 preliminary hits were obtained, which were reduced to 21 candidate molecules and subsequently to 10 unique compounds. Molecular docking identified two lead compounds, D (PubChem ID: 166484985) and I (PubChem ID: 166484904), with strong binding affinity toward the GyrB ATPase domain. ADMET and toxicity analyses indicated favorable drug-likeness and safety profiles. Molecular dynamics simulations and density functional theory studies further confirmed stable protein-ligand interactions and significant electronic reactivity.

**Discussion:**

Compounds D and I demonstrated promising ATP-competitive inhibition of the GyrB ATPase domain and exhibited distinct species-specific interaction profiles. These findings support their potential as novel anti-tubercular lead scaffolds for further experimental validation and therapeutic development.

## Graphical Abstract

**Figure:**
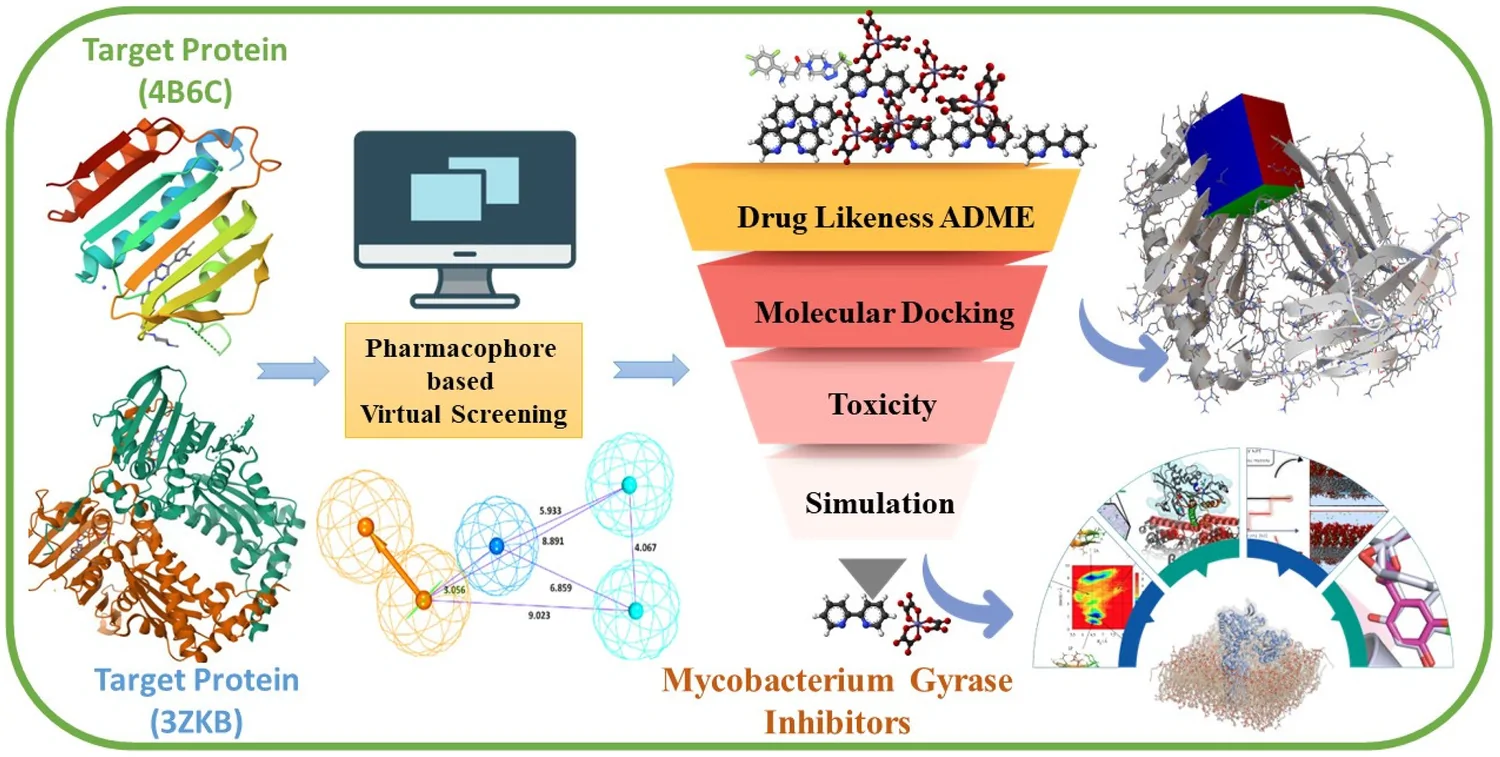

## 1 Introduction

Human tuberculosis (TB) remains a major health issue caused by the Gram-positive, acid-fast bacterium *Mycobacterium tuberculosis* (*Mtb*), which is resistant to multiple drugs (1). The World Health Organization (WHO) declared TB a global health emergency in 1993, and it continues to impact millions worldwide. The 2023 Global Tuberculosis Report reports nearly 10.8 million new cases and about 1.25 million deaths each year, with mortality roughly twice that of HIV/AIDS (2). The majority of these cases were concentrated in 30 high-burden countries, with five nations-India, Indonesia, China, the Philippines, and Pakistan-collectively contributing to about 56% of the global TB burden (3).Projections indicate that antimicrobial resistance (AMR) could cause 300 million deaths over the next 35 years and reduce global GDP by 2%–3.5%, equating to a loss of 60–100 trillion USD by 2050 (4). The increasing emergence of antimicrobial resistance (AMR), especially multidrug-resistant tuberculosis (MDR-TB), has made the management of TB more difficult and compromised the efficacy of current therapeutic regimens (5). The standard first-line anti-tubercular regimen includes rifampicin, isoniazid, pyrazinamide, and ethambutol. Among these, Pyrazinamide (PZA) is an essential first-line anti-tubercular drug whose therapeutic activity depends on its conversion by the enzyme pyrazinamidase (Pzase) into the active metabolite pyrazinoic acid (POA). The generated POA disrupts mycobacterial membrane energetics and intracellular homeostasis, ultimately contributing to the death of Mycobacterium tuberculosis cells (6, 7).

However, the widespread emergence of MDR-TB strains resistant to rifampicin and isoniazid has become a major challenge in TB control. MDR Mtb strains primarily arise from chromosomal mutations in drug-resistance-associated genes, resulting in altered drug targets, impaired prodrug activation, and reduced therapeutic susceptibility (8). Over 96% of resistant *Mtb* strains carry mutations in the 81 bp region of the rpoB gene, which encodes the RNA polymerase beta subunit, or in the 16S rRNA gene (9). Additionally, the unique lipid-rich mycobacterial cell wall and slow bacterial growth contribute significantly to intrinsic resistance and persistence (10).

DNA gyrase is a bacterial type II topoisomerase that serves as a proven therapeutic target for antibacterial drug development (11). Unlike most bacterial species, which harbour both DNA gyrase and topoisomerase IV, *Mtb* relies exclusively on DNA gyrase to carry out the critical cellular processes ordinarily mediated by both enzymes (12). It performs the dual functions of negative DNA supercoiling and decatenation, both of which are essential for proper DNA replication and segregation (13). The enzyme exists as an A₂B₂ heterotetramer composed of GyrA and GyrB subunits, where GyrA mediates DNA cleavage and rejoining, whereas the GyrB subunit hydrolyses ATP to provide the energy required for negative DNA supercoiling and other essential DNA topological processes (14). Favourable therapeutic outcomes have been observed with fluoroquinolone-containing regimens in the treatment of clinical tuberculosis (15). While typical fluoroquinolones target the cleavage-reunion core of the GyrA subunit, rising clinical resistance due to mutations in the GyrA quinolone resistance-determining region has shifted therapeutic emphasis toward the enzyme's energetic engine, the GyrB subunit (16). High-level resistance to fluoroquinolones in strains of *Mtb* and *Msm (Mycobacterium Smegmatis)* has been attributed to amino-acid substitutions within the predicted fluoroquinolone-binding region of the GyrA subunit encoded by the gyrA gene (17). These limitations have shifted attention toward alternative functional regions of DNA gyrase, particularly the ATPase domain of the GyrB subunit, as a promising strategy to overcome fluoroquinolone-associated resistance mechanisms.

Bacterial DNA gyrase lacks redundancy, making it an effective and appealing target for developing new drugs to fight antimicrobial resistance. Although less studied, the ATPase domain of GyrB is important in DNA metabolism and is increasingly targeted by inhibitors. Importantly, DNA gyrase is absent in humans and possesses significant structural and functional differences relative to human topoisomerases, making the GyrB ATPase domain an attractive and selective target for antibacterial drug discovery with reduced risk of off-target effects on human DNA processing enzymes (18). Moreover, the absence of a compensatory topoisomerase IV pathway in Mtb further boosts the bactericidal effect of GyrB-targeted inhibitors and identifies the ATPase domain as an intriguing target for anti-tubercular drug discovery (19). To combat antimicrobial resistance (AMR), researchers are exploring multiple strategies, including structure-based drug design and computational drug discovery, to identify novel inhibitors. These in-silico techniques significantly accelerate drug discovery by enabling virtual screening and interaction analysis of large compound libraries with biological targets (20). Currently, Novobiocin, a proven and effective antibiotic, is a natural product based on coumarin that primarily targets GyrB (21). In recent decades, the GyrB subunit of Mtb has gained attention in drug discovery, with most inhibitors blocking its ATPase activity to disrupt bacterial replication (22). Structurally, the N-terminal ATPase domain of GyrB has a highly conserved GHKL-type ATP-binding pocket; small-molecule inhibitors targeting this pocket lock the enzyme in an inactive, non-functional conformation, so freezing the catalytic cycle and circumventing GyrA-mediated resistance (19). Although DNA Gyrase B has been explored as an antibacterial target, the development of clinically successful ATPase-targeting inhibitors remains limited. Classical aminocoumarin inhibitors such as novobiocin exhibit limitations associated with toxicity, resistance, and unfavourable pharmacokinetic properties, thereby restricting their broader therapeutic applicability (23). Consequently, the identification of structurally distinct ATPase-targeting chemotypes with favourable pharmacokinetic properties continues to represent an important objective in anti-tubercular drug discovery.

To better contextualize the therapeutic relevance of our approach, the present study specifically focused on the conserved N-terminal ATPase domain of GyrB from both *Mtb* and *Msm*. *Msm* (*M. smegmatis*) is widely used as a fast-growing, non-pathogenic surrogate model for mycobacterial drug discovery because of its genetic tractability, safer handling conditions, and substantial structural similarity to pathogenic mycobacteria (24). Comparative sequence analyses reported in previous studies indicate that the ATPase domains of GyrB from *Mtb* and *Msm* exhibit high sequence conservation, particularly within the ATP-binding catalytic residues and nucleotide-interacting motifs. Structural superposition studies have further demonstrated strong conservation of the ATP-binding cavity architecture between the two species, thereby supporting the translational relevance of comparative inhibitor screening approaches.

Therefore, this study used an integrated computational workflow of structure-based pharmacophore modelling, virtual screening, molecular docking, ADMET analysis, molecular dynamics simulations and DFT calculations to identify novel GyrB inhibitors candidates targeting ATPase against *Mycobacterium* species. Energy-optimized structure-based pharmacophore models were constructed to capture key interaction features within the GyrB active site and were subsequently employed to screen a curated compound library. The resulting hits were further prioritized using stringent drug-likeness and physicochemical filtering criteria to ensure favourable pharmacokinetic properties. In parallel, ligand-based pharmacophore models specific to GyrB inhibitors of both species were developed to complement and validate the screening strategy (25). These complementary pharmacophore models can be used to identify and prioritize effective and selective inhibitors of the GyrB subunit. Together, they provide a reliable computational approach that supports the discovery of promising anti-mycobacterial candidates for further experimental validation.

## 2 Material and methods

### 2.1 Pharmacophore model generation and validation

A pharmacophore represents the essential steric and electronic features required for optimal interaction between a biologically active ligand and its target binding site. In the present study, structure-based pharmacophore models were generated from the conserved ATP-binding pocket located within the N-terminal ATPase domain of the DNA Gyrase B (GyrB) subunit of *Mtb* and *Msm*. These pharmacophoric features were defined based on the complementary interactions between co-crystallized ligands and the active-site residues responsible for biological activity (26). The crystal structures of the GyrB ATPase domain from *Mtb* and *Msm* were retrieved from the RCSB Protein Data Bank (PDB) in PDB format. The selected structures included *Mtb* GyrB complexed with AMPPNP (27) (PDB ID: 3ZKB; resolution: 2.90 Å) and *Msm* GyrB complexed with aminopyrazinamide (28) (PDB ID: 4B6C; resolution: 2.20 Å). These structures were visualized and analyzed using the Discovery Studio Visualizer to identify critical intermolecular interactions between the co-crystallized ligands and the ATP-binding pocket residues.

Structure-based pharmacophore models were subsequently generated using the Pharmit web server. Key interacting pharmacophoric features, including hydrogen bond acceptors (HA), hydrogen bond donors (HD), aromatic ring centers (A), and hydrophobic regions (HY), were identified and spatially mapped within the active site. The generated models were further validated by evaluating the geometric arrangement and inter-feature distances of the pharmacophoric elements to ensure accurate representation of the ATPase-binding cavity.

**Figure 1:**
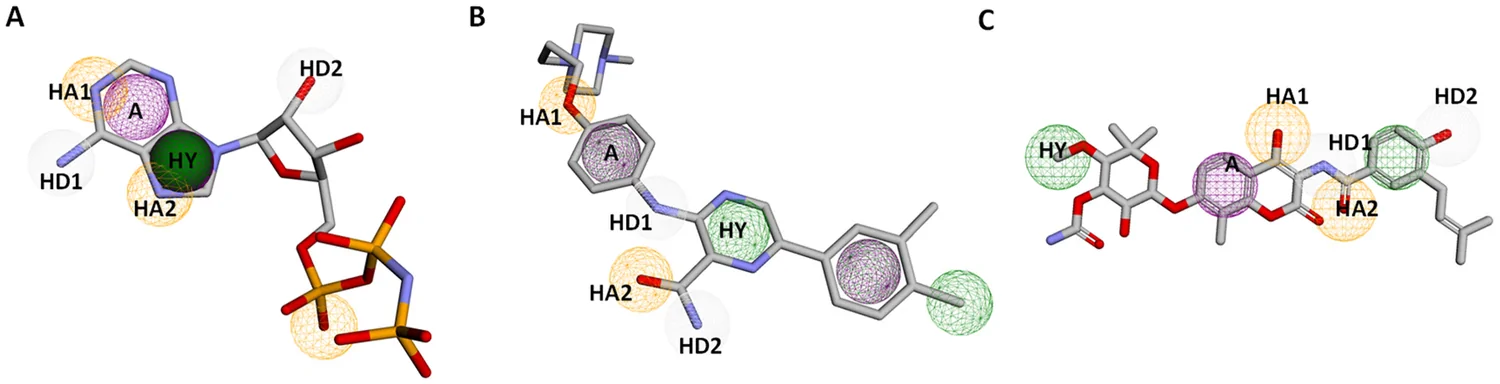
**(A)** structure based pharmacophore model generated from the ATP-binding pockets of the DNA gyrase B ATPase domains of **(A)** *Mtb*(PDB ID: 3ZKB) and **(B)** *M. smegmatis* (PDB ID: 4B6C) **(C)** Novobiocin [A: Aromatic; HY: Hydrophobic; HA: Hydrogen-bond acceptor; HD: Hydrogen-bond donor].

To assess the predictive reliability of the developed pharmacophore models, the established ATP-competitive GyrB inhibitor novobiocin was mapped onto the generated pharmacophoric features. Novobiocin exhibited favorable alignment with the pharmacophore models by satisfying the essential hydrogen bond donor/acceptor, aromatic, and hydrophobic interaction requirements associated with ATPase-pocket recognition. The finalized pharmacophore models for 3ZKB and 4B6C are illustrated in Figure 1, while the corresponding pharmacophoric features are summarized in Table 1.

**Table 1:** Inter-feature distance(Å) between pharmacophoric elements of 3ZKB & 4B6C used for pharmacophore model generation.

Pharmacophoric features	3ZKB						4B6C					
	HD1	HD2	HA1	HA2	A	HY	HD1	HD2	HA1	HA2	A	HY
HD1	-	7.03	4.97	1.80	3.75	3.14	5.57	10.11	-	7.72	2.79	8.12
HD2	7.03	-	5.35	4.24	4.63	3.53	2.56	2.28	7.72	-	4.79	3.54
HA1	4.97	5.35	-	3.77	1.49	3.36	-	4.76	5.57	2.56	2.78	2.50
HA2	1.80	4.24	3.77	-	2.45	1.14	4.76	-	10.11	2.28	7.05	3.20
A	3.75	4.63	1.49	2.45	-	2.17	2.78	7.05	2.79	4.79	-	5.63
HY	3.14	3.53	3.36	1.14	2.17	-	2.50	3.20	8.12	3.54	5.63	-

### 2.2 Pharmacophore-based virtual screening

Pharmacophore-based virtual screening was performed using the Pharmit web server (http://pharmit.csb.pitt.edu/), which integrates several publicly accessible chemical libraries, including PubChem, ZINC, MolPort, ChemSpace, ChEMBL34, MCULE, and LabNetwork (29). Structure-based pharmacophore models generated from the ATP-binding pockets of the DNA Gyrase B ATPase domains of *Mtb* (PDB ID: 3ZKB) and *M. smegmatis* (PDB ID: 4B6C) were employed as pharmacophore queries to identify structurally diverse compounds with potential inhibitory activity.

Virtual screening was initially conducted against approximately one million compounds compiled from multiple chemical databases. Compounds satisfying the essential pharmacophoric constraints, including hydrogen bond donor/acceptor features, aromatic ring interactions, and hydrophobic moieties complementary to the ATPase active site, were retained for further analysis. The screening process yielded approximately 30,000 preliminary hits, primarily from the PubChem database. To minimize false-positive candidates and improve drug-likeness, the retrieved compounds were subjected to sequential filtering based on Lipinski's Rule of Five, physicochemical property evaluation, and preliminary ADMET prediction. Parameters including molecular weight, hydrogen bond donor and acceptor counts, lipophilicity, and predicted pharmacokinetic behavior were considered during compound selection. Based on these criteria, 21 compounds demonstrating favorable drug-like characteristics were shortlisted for molecular docking studies.

**Figure 2:**
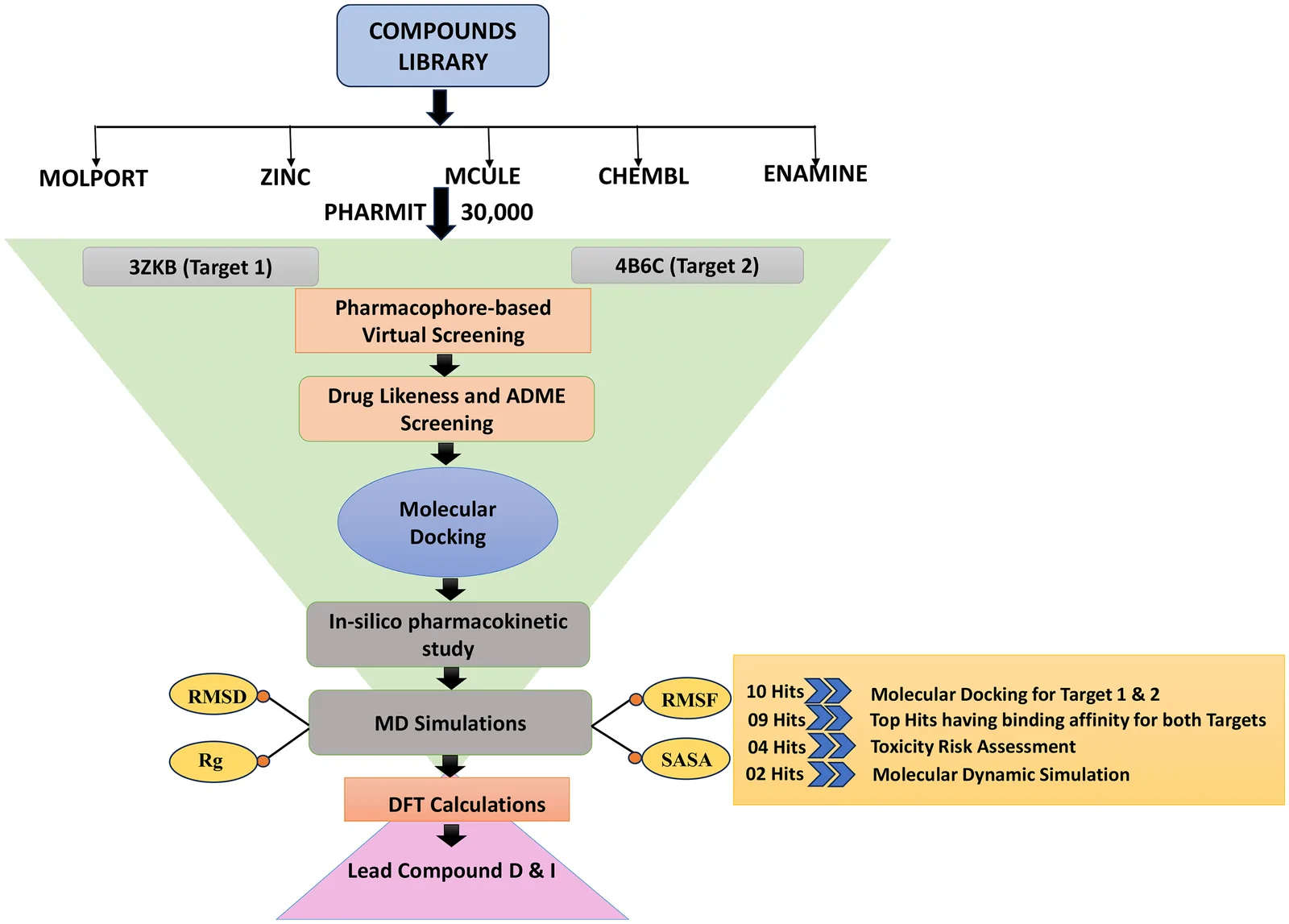
In-silico workflow for the identification of potential DNA gyrase B inhibitors against *Mtb* (3ZKB) and *Msm*(4B6C).

The shortlisted compounds were subsequently docked into the ATP-binding pocket of the GyrB ATPase domain. Final selection of ten lead compounds was based on docking score, binding orientation, interactions with key catalytic residues, structural diversity, and RMSD-based conformational stability. Compounds exhibiting redundant scaffolds or highly similar binding conformations were excluded to retain structurally unique candidates. The selected hits were further subjected to detailed interaction analysis, molecular dynamics simulations, and density functional theory (DFT) calculations to identify the most promising lead molecules. The overall computational workflow employed in this study is illustrated in Figure 2.

### 2.3 Drug-likeness and ADME analysis

Drug likeness and Absorption, Distribution, Metabolism, and Excretion (ADME) properties of the pharmacophores were analysed using Swiss ADME (30). These evaluation criteria served as basis for filtering the virtual ligands. Specifically, violations of Lipinski rule of five, Veber's criteria and Ghose filters were assessed, along with blood-brain barrier permeability, to effectively sxreen and identify promising lead compounds.

### 2.4 Target protein preparation

The crystal structures of the Gyrase B target proteins for *Mtb* (3ZKB) and *Msm* (4B6C) were visualized using Discovery Studio. Their x-ray crystallography structures were obtained from the RCSB PDB database. Prior to molecular docking, these proteins were prepared by removing water molecules, heteroatoms, and co-crystallized inhibitors. Energy minimization was then performed using AutoDock software.

### 2.5 Lead optimization

The top lead molecules from the library, along with their PubChem IDs, were retrieved from the Pharmit server. These lead molecules were subjected to energy minimization using ChemDraw Ultra. Further molecular docking was performed using AutoDock software.

### 2.6 Grid preparation

Grid preparation was carried out in AutoDock using the co-crystallized ligand and the reported literature to select the amino acid residues. The co-crystallized ligand with the target protein was analysed using Discovery Studio Visualizer. Grid preparation for the PDB ID 3ZKB was set up by selecting the amino acid residues Asn 52, Leu 120, His 121, Val 123, Gly 122, Gln 370, Gly 124, Val 125, Gly 107, Tyr 12, Asp 79, Lys 372, Ile 17, Tyr 114 (27). The grid box with X, Y, Z parameters was set to 80, 126, 104 with a grid spacing of 0.375 Å. Grid box preparation for the PDB ID 4B6C involved selecting amino acid residues based on the literature and co-crystallized ligand as follows: Asp 79, Arg 82, Arg 141, Ile 171, Val 49, Val 128, Val 123, Val 99, Phe 199, Ile 84, Asn 52, Tyr 253, Asp 97, Pro 85, Gly 83. The grid box with X, Y, Z parameters was set up to 97, 113, 69 with a grid spacing of 0.375 Å (28).

### 2.7 Toxicity analysis

The toxicity profiles of the screened compounds (A–J) were evaluated using the ADMET lab 3.0 web server. Key toxicity parameters, including Ames mutagenicity, cytotoxicity, and carcinogenicity, were evaluated to assess the safety profile of the selected compounds (31). The predicted toxicity data were further used to identify potential lead molecules with favourable toxicity characteristics.

### 2.8 Sequence alignment and in silico analysis

The amino acid sequences of DNA gyrase subunit B (GyrB) from *Mtb* and *Msm* were analyzed through both multiple and pairwise sequence alignments to assess sequence conservation, domain differences, and calculate sequence metrics. MUSCLE, with default settings, was employed for multiple sequence alignment (MSA) to accurately position conserved residues and define domain boundaries (32). The final alignment was visualized using the ESPript 3.0 server, which highlighted secondary structural elements, conservation patterns, and identical residues (33). Additionally, the crystal structure of the mycobacterial GyrB ATPase domain (PDB ID: 3ZKB) served as a structural template in ESPript to precisely map secondary structures within the core functional region (∼1–430 aa). Global pairwise sequence alignment was carried out utilizing the EMBOSS Needle online tool via the EMBL-EBI framework for a thorough quantitative comparison (34). Using a BLOSUM62 substitution matrix, a gap-opening penalty of 10.0, and a gap-extension penalty of 0.5, this alignment applied the Needleman-Wunsch method under default scoring parameters (35).This ideal pairwise alignment was then used to determine the exact percentages of global sequence identity and similarity.

### 2.9 Homology modeling and structural superimposition

The SWISS-MODEL tool was used to generate homology models of the DNA gyrase subunits from *Mtb* and *Msm*. High sequence similarity and comprehensive structural coverage were ensured by selecting the best structural templates from the best-fit AlphaFold structures (36). UCSF ChimeraX was used to examine the three-dimensional structures of the gyrase B proteins of *Mtb* and *Msm* and to assess structural variations between them (37). The matchmaker command, which aligns sequences using BLOSUM62 and iteratively superimposes the C*α* backbone atoms in three-dimensional space, was used to perform structural superimposition. The RMSD of the aligned C*α* atom coordinates was used to quantify the structural similarities between these enzymes.

### 2.10 Molecular docking

Molecular docking was performed using AutoDock software, focusing on the active site of the target protein the Gyr B subunit ATPase domain of both 3ZKB and 4B6C, employing the Lamarckian Genetic Algorithm (38). Initially, each ligand was docked with the active site of the target proteins. The number of runs was set to 100. AutoDock Tools (ADT) were used to generate the grid and docking parameter files in the gpf and dpf formats. The ligands with the best binding affinity, exhibiting non-covalent interactions, were visualised using Discovery Studio Visualizer software. Docking studies were also conducted using the dimeric ATPase structures of DNA Gyrase B (3ZKB and 4B6C; see Supplementary Table & Figures S1 & S2). The selected promising compounds demonstrated binding modes and key ATP-pocket interactions comparable to those observed in the monomeric ATPase domain. However, the dimeric structures exhibited relatively lower docking scores than their monomeric counterparts. No major differences were found in the ATP-binding residues and ligand interaction pattern between the two systems. The monomeric ATPase domain was selected for further computational analyses, as it retained the critical active-site architecture and gave more favourable docking scores with more clear ligand-residue interaction profiles.

### 2.11 Molecular dynamics simulation

The two promising compounds, D and I, which exhibit high affinity for the proteins 3ZKB and 4B6C, respectively, were selected for molecular dynamics (MD) simulations to assess and compare the stability of their respective protein-ligand complexes over time. Molecular dynamics (MD) simulations were conducted for 100 ns using the Desmond module of the Schrödinger Suite (2020-3) following standard protocols (39, 40). Each protein-ligand complex was prepared as a solvated system using the System Builder panel in Desmond, employing the OPLS3e force field, which is recognised for its enhanced accuracy in modelling small molecules and proteins (41). For both proteins, an orthorhombic box was constructed utilising the Single Point Charge (SPC) solvent model, which is essential for accurately simulating the solvation environment (42). To ensure system neutrality, counterions (Na + and Cl−) were randomly added, and a 0.15 M NaCl solution was included to maintain an isosmotic environment, reflecting physiological conditions (39). Following system setup, energy minimisation was performed according to Desmond's standard protocol, utilising the OPLS3e force field parameters to optimise the geometry of the solvated systems. After energy minimisation, the system was relaxed and prepared for a production run lasting 100 ns. During this simulation, 1000 frames were captured for subsequent analysis. The Simulation Interaction Diagram (SID) tool was employed to analyse the trajectory data from the MD simulations, allowing for a detailed evaluation of the complexes' stability. This analysis included assessing key metrics such as root-mean-square deviation (RMSD) and root-mean-square fluctuation (RMSF), radius of gyration (Rg), and solvent-accessible surface area (SASA), as well as hydrogen-bond interaction analysis which are essential for understanding the dynamics and stability of protein-ligand interactions.

The binding free energy (Gbind) of the docked complexes was determined with the premier MM-GBSA module (Schrodinger suite, LLC, New York, NY, USA, 2017-4). Calculations employed the OPLS 2005 force field, the VSGB solvent model, and rotamer-search methods (43–45). Post-MD, 10 ns interval frames from the trajectory were selected. The overall free energy binding was computed using Equation (1):

ΔGbind=Gcomplex−(Gprotein+Gligand)

where *Δ*Gbind is the binding free energy, Gcomplex is the free energy of the complex, Gprotein is that of the target protein, and Gligand is that of the ligand. The resulting MM-GBSA trajectories were further analyzed for structural modifications after the dynamics.

### 2.12 Density functional theory (DFT) analysis

To investigate the electronic structures and properties of selected ligands, we have carried out DFT analysis using Gaussian 16, Revision B.01 suite (46). Initially, all drugs were optimized using B3LYP/ 6-31G** level of theory (47, 48). B3LYP functional method and exchange–correlation functional with a 6-31G** basis set have been used for carbon, nitrogen, oxygen, and hydrogen atoms. Relevant energetic properties, such as electronic energy and Zero Point Energy (ZPE), were calculated for each ligand. All Figures have been made by Chemcraft (http://www.chemcraftprog.com).

## 3 Result & discussion

### 3.1 Pharmacophore-based virtual screening

The pharmacophores were developed based on both the target protein Gyrase B ATPase subunit (PDB ID: 3ZKB) and the 4B6C complexed with AMPPNP and Aminopyrazinamide respectively. The pharmacophore features, including hydrogen-bond donor, hydrogen-bond acceptor, aromatic ring, and hydrophobic ring, were derived from the ligand-receptor complexes. A total of 21 hits were identified after filtering through Lipinski's rule. Of these, 10 hits with different PubChem IDs, differed in their RMSD values; hence, all 10 hits were subjected to molecular docking. The encompassing sphere, which included all features from the models, was used to perform the pharmacophore-based virtual screening. The best results were obtained from hits displaying binding affinity for both targets.

### 3.2 Drug likeness and ADME screening

**Figure 3:**
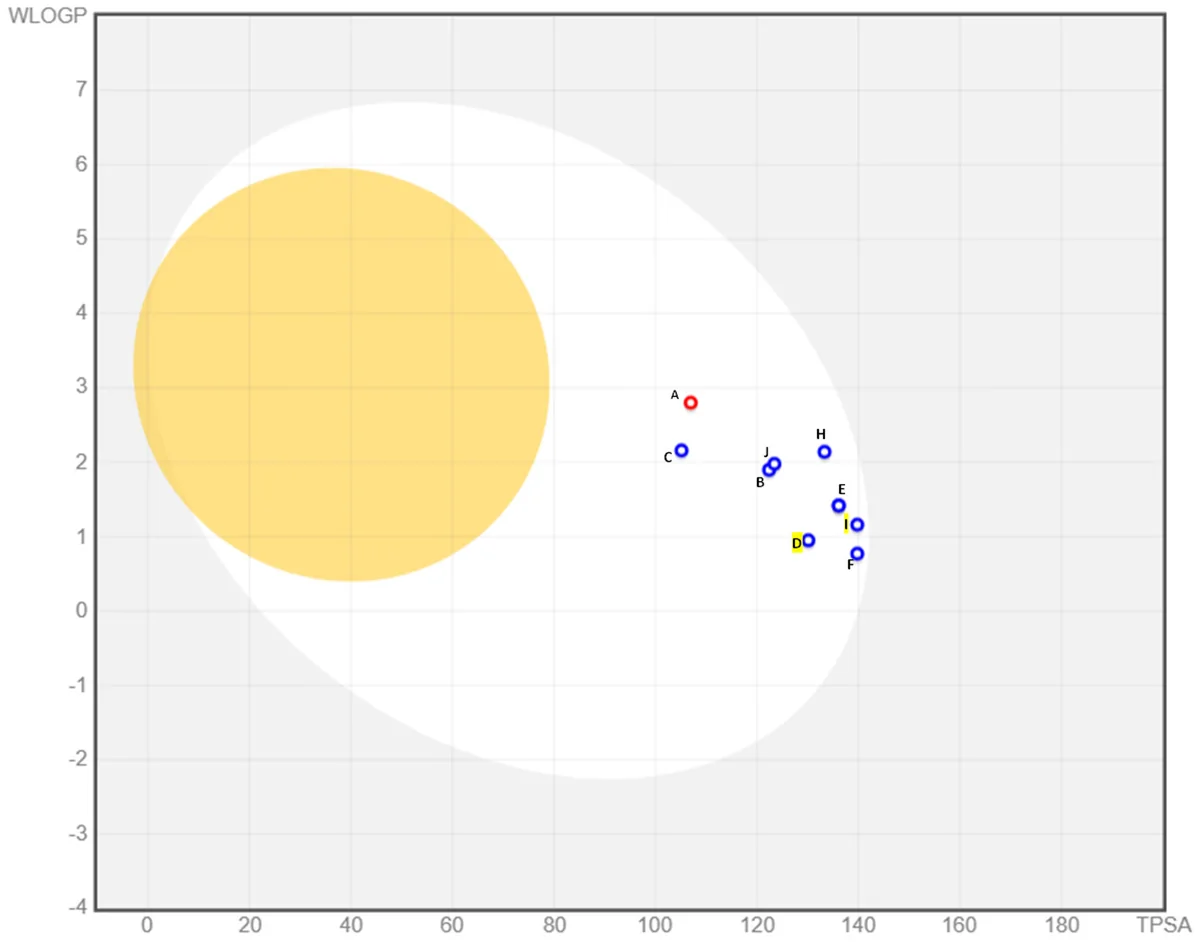
Boiler-Egg model prediction of gastrointestinal absorption and brain penetration of compounds **(A–J)** along with the reference drug novobiocin.

The better hits obtained from both target proteins were evaluated for physicochemical and drug-likeness properties. The physicochemical and ADME properties of the ligands, analysed using SwissADME, are presented in the Table 2. SwissADME's BOILED-Egg model was used to assess the pharmacokinetic characteristics of the designed compounds. The model uses lipophilicity (WLOGP) and topological polar surface area (TPSA) to predict passive gastrointestinal (GI) absorption and blood–brain barrier (BBB) penetration. The bulk of the identified compounds (A-J) were found in the egg's white region, as shown in Figure 3, suggesting a high likelihood of good gastrointestinal absorption. For non-CNS antibacterial agents, limited BBB penetration is preferred, as none of the compounds were found in the yellow yolk region. According to its established pharmacokinetic profile, the reference medication novobiocin also showed up outside the BBB region. Overall, the BOILED-Egg analysis indicates that the suggested molecules have low central nervous system penetration and favourable oral bioavailability. Novobiocin and our screen compound G were predicted to be outside the BOILED-Egg applicability domain, likely due to their high molecular weight and elevated polar surface area, which together indicate limited passive gastrointestinal absorption and negligible blood-brain barrier permeability. The colour coding of compounds denotes their interaction with P-glycoprotein, where blue circles correspond to predicted P-gp substrates and red circles to non-substrates, influencing cellular retention and permeability.

**Table 2:** Physicochemical and pharmacokinetic analysis of the screened compounds.

S. No	PubChem Id	Molecular Weight	Aromaticity	Rotatable bonds	H bond donor	H bond acceptor	TPSA	LogP	BBB Permeation	Lipinski violation	Egan Violation	Muegge Violation	PAINS #alert	BRENK #alert
1	126515938	388.38	23	4	2	6	106.95	2.38	No	0	0	0	0	0
2	71597198	480.54	18	9	2	9	122.39	3.84	No	0	0	0	0	0
3	161135710	446.5	21	5	2	5	105.14	0	No	0	0	0	0	0
4	166484985	486.57	21	7	3	7	130.12	3.17	No	1	0	0	0	0
5	166484747	485.54	21	7	3	7	136.11	2.7	No	1	1	0	0	0
6	166484853	430.47	21	5	3	7	139.77	1.79	No	1	1	0	0	0
7	166484709	473.53	21	7	3	7	136.11	2.39	No	1	1	0	0	0
8	166484986	492.51	21	7	2	9	133.31	2.9	No	1	1	0	0	0
9	166484904	444.49	21	6	3	7	139.77	1.91	No	1	1	0	0	0
10	66604907	431.44	19	5	2	6	123.43	2.68	No	0	0	0	0	0

### 3.3 Toxicity analysis

Parameters including Lipinski's rule compliance, gastrointestinal absorption, blood–brain barrier permeability, cytochrome P450 inhibition, hepatotoxicity, Ames mutagenicity, carcinogenicity, and bioavailability were considered during lead prioritization. Compounds exhibiting unfavorable toxicity predictions, poor pharmacokinetic profiles, or multiple drug-likeness violations were deprioritized.The majority of the compounds were predicted to be non-mutagenic and non-cytotoxic, indicating a favourable safety profile. Compounds B, D, G, H, I, and J showed no Ames toxicity, cytotoxicity, or carcinogenicity, suggesting their suitability for further drug development. In particular, compounds D and I, which also demonstrated superior docking scores and stability in molecular dynamics simulations, exhibited a completely non-toxic profile across all evaluated parameters.

However, certain compounds displayed potential toxicity concerns. Compounds C and F were predicted to be Ames positive, indicating possible mutagenic effects, while compounds A, E, and F showed carcinogenic tendencies. Although these compounds were non-cytotoxic, the presence of mutagenicity or carcinogenicity alerts may restrict their further progression without structural optimization. The top hits with zero Lipinski, Veber, and Muegge violations were further subjected to toxicity analysis, as shown in Table 3.

**Table 3:** *In- silico* toxicity prediction of screened compounds.

S. No	Compound Id	PubChem Id	Ames toxicity	Cytotoxicity	Carcinogenicity
1	A	126515938	Inactive	Inactive	Active
2	B	71597198	Inactive	Inactive	Inactive
3	C	161135710	Active	Inactive	Inactive
4	D	166484985	Inactive	Inactive	Inactive
5	E	166484747	Inactive	Inactive	Active
6	F	166484853	Active	Inactive	Active
7	G	166484709	Inactive	Inactive	Inactive
8	H	166484986	Inactive	Inactive	Inactive
9	I	166484904	Inactive	Inactive	Inactive
10	J	66604907	Inactive	Inactive	Inactive

Overall, the toxicity assessment highlights compounds D and I as the most promising candidates, combining favourable safety profiles with strong binding affinity and structural stability, thereby supporting their selection for further experimental validation.

### 3.4 Sequence and structural model analysis

**Figure 4:**
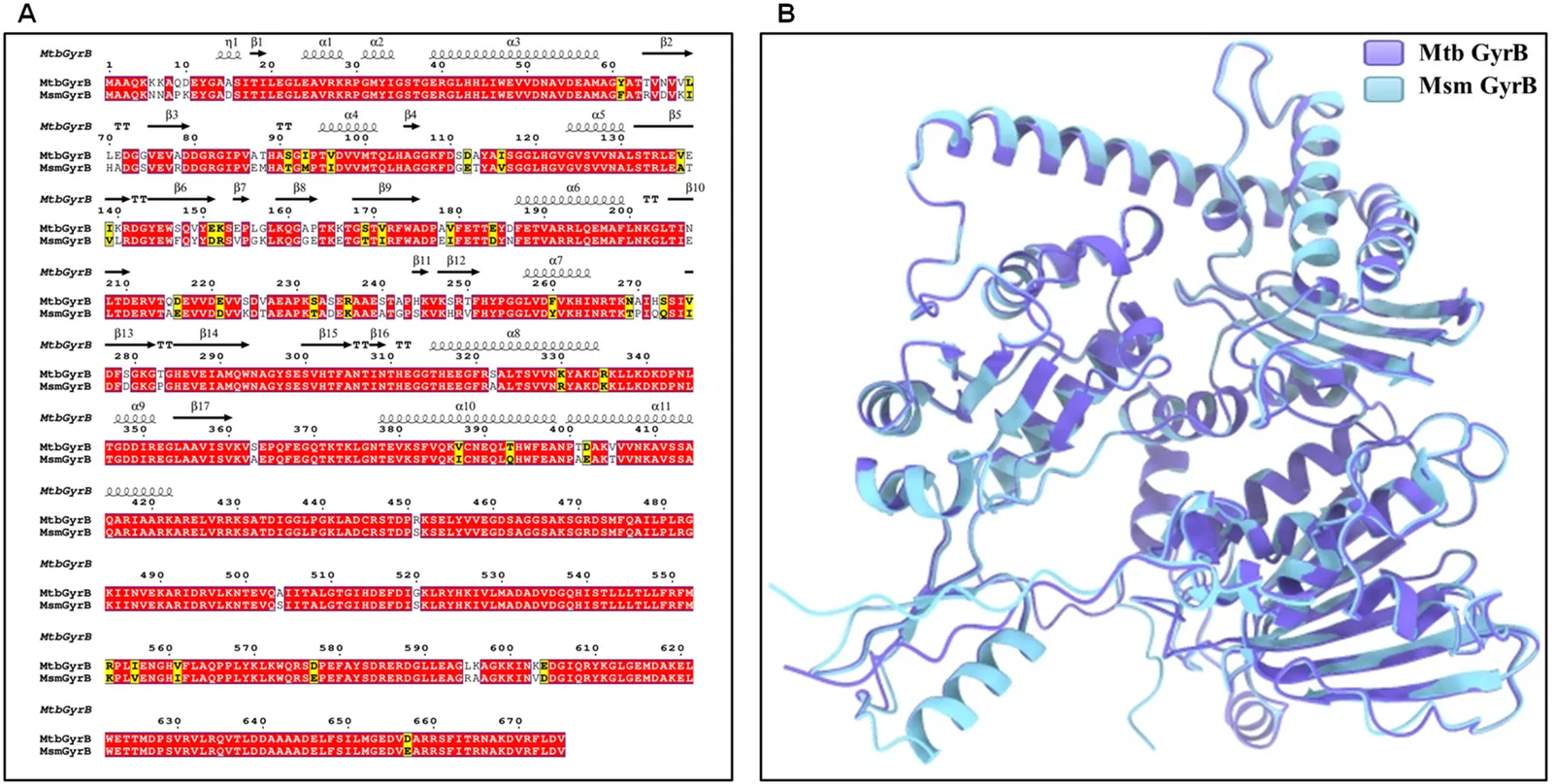
**(A)** sequence alignment of *Mtb* gyrB & *Msm* gyrB highlighting conserved amino acid residues. **(B)** Superimposed Structural model of *Mtb* and *Msm* GyrB Proteins showing high structural similarity.

The sequence alignment of GyrB from both *Mtb* and *Msm* reveals a high conservation of residues 1–675 (Figure 4A). The full-length GyrB proteins of *Mtb* and *Msm* are each exactly 675 amino acids long. They share 87.3% sequence identity and 92.7% similarity across their entire length. The residue numbering within the active-site ATPase pocket (positions 1–430) is also identical in both species. The ATPase domain exhibits 82.8% identity and 89.8% similarity. Structural differences were evaluated by superimposing the three-dimensional homology models of *Mtb* GyrB and *Msm* GyrB using UCSF ChimeraX (Figure 4B). The alignment demonstrated an exceptional level of structural conservation, with a global RMSD of 0.365 Å over the aligned C*α* backbone atoms. This minor deviation confirms that the high sequence similarity results in a nearly identical tertiary structure, with consistent secondary structural elements in both species. Consequently, this structural similarity supports employing *Msm* as a surrogate model for initial in silico screening and lead development.

In structure-based drug design, this high similarity enables lead compounds to target the same hydrogen-bonding and hydrophobic regions, reducing the risk of structural mismatches and ensuring effectiveness against both drug-susceptible and MDR Mtb strains.

### 3.5 Molecular docking

Molecular docking is the most effective computational technique for virtually screening for optimal interactions between a ligand and the target protein's active-site binding pocket, based on its docking score and reported hydrogen bonds and other significant interactions (e.g., alkyl, van der Waals, electrostatic, hydrophobic). Initially, we identified 21 hits against *Mtb* and 8 hits against *Msm* gyrase protein after filtering with Lipinski's rule using Pharmit software. The compounds selected for docking studies against the target proteins of both organisms were retrieved from the PubChem database. The corresponding compound IDs are 126515938, 71597198, 161135710, 166484985, 166484747, 166484853, 166484709, 166484986, 166484904, and 66604907, along with the reference standard novobiocin (PubChem ID: 54675769). These compounds are illustrated in Figure 4. Compounds PubChemA (126515938), PubChemD (166484985), and PubChemI (166484904) demonstrated the highest binding affinities toward the GyrB subunit of *Mtbs* (PDB ID: 3ZKB), with docking scores of −10.07, −10.22, and −10.16 kcal/mol, respectively, indicating strong and stable ligand–protein interactions within the active site.

In the case of *Msm* (PDB ID: 4B6C), multiple compounds, including PubChemA (126515938), PubChemB (71597198), PubChemC (161135710), PubChemD (166484985), PubChemE (166484747), and PubChemJ (66604907), exhibited favourable binding profiles, with docking scores ranging from −10.34 to −12.18 kcal/mol. Notably, these compounds formed key hydrogen-bond interactions with active-site residues, suggesting enhanced binding stability and specificity toward the GyrB subunit.

The reference ligand, novobiocin, showed comparatively lower binding affinity against *Mtb*GyrB (−9.65 kcal/mol), despite forming five hydrogen bonds, indicating that factors beyond hydrogen bonding, such as hydrophobic and van der Waals interactions, may contribute significantly to overall binding stability. Furthermore, novobiocin exhibited substantially weaker binding to *M. smegmatis* GyrB (−3.36 kcal/mol), despite forming four hydrogen bonds, highlighting a marked difference in binding efficiency relative to the screened compounds.

Compound PubChemA (126515938) formed two hydrogen bonds with residues Thr189 and Arg193 in the *Mtb*GyrB protein (PDB ID: 3ZKB), exhibiting a docking score of −10.07 kcal/mol. In the *Msm* GyrB protein (PDB ID: 4B6C), it established three hydrogen bonds with Asp79, Asp79, and Arg82, achieving the highest docking score of −12.18 kcal/mol, indicating strong binding stability.

Compound PubChemB (71597198) formed two hydrogen bonds with Arg40 and Arg193 in 3ZKB, with a docking score of −9.28 kcal/mol, while in 4B6C, it exhibited four hydrogen bonds with Thr169, Asp79, Asp79, and Arg141, with a docking score of −10.40 kcal/mol. Compound PubChemC (161135710) established three hydrogen bonds with Asp309, Thr310, and Thr303 in 3ZKB, and with Arg141, Asp79, and Asn52 in 4B6C, showing docking scores of −9.48 kcal/mol and −10.96 kcal/mol, respectively.

Compound PubChemD (166484985) formed three hydrogen bonds (Asn309, Arg40, Arg40) with 3ZKB, yielding a docking score of −10.22 kcal/mol, and two hydrogen bonds (Gln102, Glu48) with 4B6C, with a docking score of −11.85 kcal/mol. Compound PubChemE (166484747) formed a single hydrogen bond with Asn309 in 3ZKB (−8.69 kcal/mol) and one with Asp79 in 4B6C (−10.34 kcal/mol).

Compound PubChemF (166484853) formed one hydrogen bond with Asn309 in 3ZKB, with a docking score of −9.78 kcal/mol, while in 4B6C it formed two hydrogen bonds with Asn52 and Asn52, with a docking score of −9.30 kcal/mol. Compound PubChemG (166484709) exhibited four hydrogen bonds (Arg40, Trp47, Asn309, Glu196) with 3ZKB, with a docking score of −9.65 kcal/mol; however, despite forming three hydrogen bonds (Glu48, Asn52, Gln102) with 4B6C, it showed an anomalously high docking score of +124 kcal/mol, indicating an unfavorable interaction.

Compound PubChemH (166484986) formed three hydrogen bonds (Glu196, Arg40, Trp47) with 3ZKB, with a docking score of −8.55 kcal/mol, and one hydrogen bond (Val49) with 4B6C, with a docking score of −9.46 kcal/mol. Compound PubChemI (166484904) established three hydrogen bonds involving Arg40, Arg40, and Arg40 in 3ZKB, with a favourable docking score of −10.16 kcal/mol, and two hydrogen bonds (Asn52, Ser126) in 4B6C, with a docking score of −9.34 kcal/mol.

Compound PubChemJ (66604907) formed three hydrogen bonds (His43, Glu196, Glu196) with 3ZKB, resulting in a docking score of −9.61 kcal/mol, and multiple hydrogen bonds (Asn52, Arg82, Gly83, Asp79, Arg141) with 4B6C, with a docking score of −11.06 kcal/mol.

**Figure 5:**
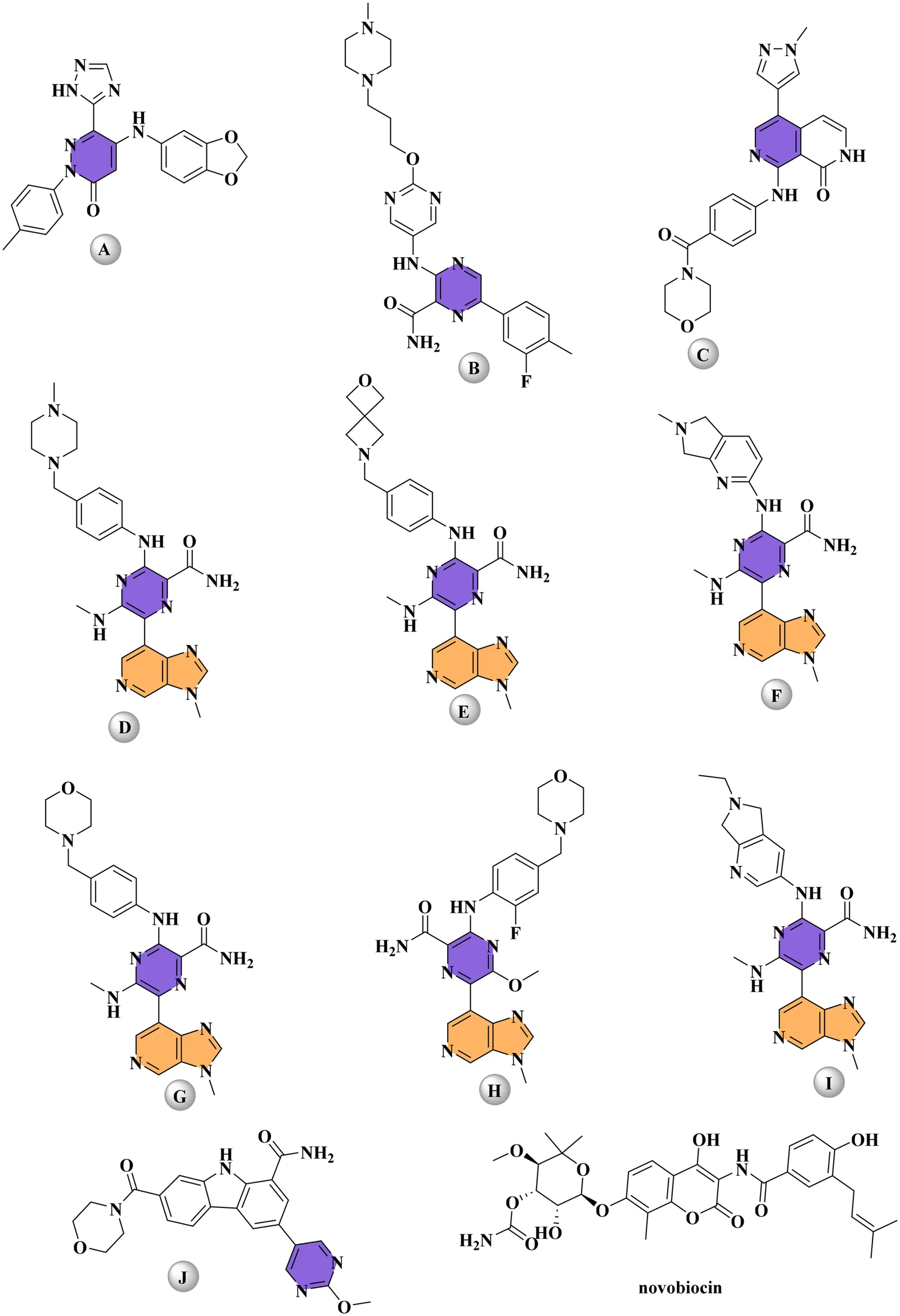
Chemical structures of screened compounds **(A–J)** selected from the pubChem database.

The docking results, including binding affinities and key residue interactions, are summarized in Table 4, while the Docking 2D and 3D interaction profiles with target proteins 3ZKB and 4B6C are presented in Figure 5.

**Table 4:** Molecular docking results of selected pharmacophore-derived compounds (A-J) and the standard drug novobiocin against the gyrB subunit of *Mtb*(PDB ID: 3ZKB) and *msms* (PDB ID: 4B6C), showing docking scores (kcal/mol), number of hydrogen bonds, and key interacting amino acid residues.

S.No	Compounds	*M. tuberculosis* (3ZKB)		*M. smegmatis* (4B6C)	
		Score (kcal/mol)	Hydrogen/ Remarkable bonds	Score (kcal/mol)	Hydrogen/Remarkable bonds
1	A	−10.07	Thr 189, Arg193(H bond), Arg 40, Arg 192, Ile 308(Alkyl), Trp 47(Pi -Pi stacked), Asn 309(Pi -Sigma).	−12.18	Asp 79, Asp 79, Arg 82 (H bond), Val 49, Val 128, Val 123, Ile 171, Val 77, Pro 85, Ile 84 (Alkyl), Thr 169(Carbon- Hydrogen bond), Glu 56(Pi Anion).
2	B	−9.28	Arg 40, Arg 193(H bond), His 302, Ile 308, Lys 372(Alkyl), Glu 299, Glu 317(Vander-waal), His 311, Trp 47, His 44(Pi -Pi stacked).	−10.40	Thr 169, Asp 79, Asp 79, Arg 141(H bond), Pro 85, Ile 84, Val 99, Ile 171, Val 128, Val 49, Ala 53(Alkyl), Ala 53, Gln 102, Glu 48 (Carbon- Hydrogen Bond), Glu 56, Arg 82(Pi Anion).
3	C	−9.48	H bond: Asn 309, Thr 310, Thr 303, Thr 303; Alkyl: Thr 303, Arg 193, Arg 192; Vander-waal: Glu 299, Asn 309.	−10.96	Arg 141, Asp 79, Asn 52(H bond), Val 128, Val 49, Val 123, Pro 85, Ile 84(Alkyl), Glu 56(Carbon- Hydrogen bond).
4	D	−10.22	Asn 309, Arg 40, Arg 40(H bond), Glu 196, Arg 193, Glu 39, Asp 186(Carbon Hydrogen bond).	−11.85	Gln 102, Glu 48 (H bond), Val 49, Ala 53, Ile 171, Ile 84(Alkyl), Val 99, Asp 79, Glu 48(Vander-waal), Glu 48(Pi Anion).
5	E	−8.69	Asn 309(H bond), Thr 303, Val 301, Glu 299, Glu 317(Carbon- Hydrogen Bond), Trp 47(Alkyl).	−10.34	Asp 79(H bond), Arg82, Pro 85, Ile 84, Val 123(Alkyl), Ile 171, Asp 79, Glu 48, Glu 48(Carbon-Hydrogen bond).
6	F	−9.78	Asn 309(H bond), Arg 40, Glu 39, Glu 39(Vander-waal), Glu 196(Salt bridge).	−9.30	Asn 52, Ser 126(H bond), Ile 171, Val 49, Ile 84, Pro 85, Arg 82(Alkyl), Glu 56, Arg 82(Pi Cation).
7	G	−9.65	Arg 40, Trp 47, Asn 309, Glu 196(H bond), His 44, Ile 34, Glu 196, Arg 192(Carbon- Hydrogen bond), His 263, Arg 193(Alkyl).	+124	Glu 48, Gln 102, Asn 52(H bond), Val 148, Asp 51, Ser 126, Asp 55(Alkyl)
8	H	−8.55	Glu 196, Arg 40, Trp 47(H bond), Arg 193, Ile 308, Trp 47, Arg 192(Alkyl), Glu 196, Arg 40, His 44, Ile 34, Trp 47(Carbon- Hydrogen bond).	−9.46	Val 49(H bond), Glu 56, Gly 83, Glu 48, Glu 48, Glu 102(Carbon- Hydrogen Bond), Val 123, Ile 84(Alkyl).
9	I	−10.16	Arg 40, Arg 40, Arg 40(H bond), Lys 372, His 44, Arg 40(Alkyl), Glu 196(Attractive charge)	−9.34	Ser 126, Asn 52(H bond), Ile 84, Ile 171, Ala 53, Val 127(Alkyl), Asp 79, Asp 79, Glu 48(Carbon- Hydrogen Bond).
10	J	−9.45	His 43, Glu 196, Glu 196(H bond), Lys 372, Ile 308, His 44, Arg 40(Alkyl), His 44(Carbon -Hydrogen bond).	−10.77	Asn 52, Arp 79, Arg 82, Gly 83, Arg 141(H bond), Pro 85, Pro 85, Ile 171, Val 49, Val 123, Val 128, Ile 84(Alkyl), Glu 56(Pi Cation).
11	Novobiocin	−9.65	Val 301, Glu 299, Arg 40, Thr 371, Glu 196(H bond).	−3.36	Lys 157, Val 86, Pro 85, Val 99(H bond).

Comparative analysis indicates that compounds PubChemA (126515938), PubChemB (71597198), PubChemC (161135710), PubChemD (166484985), PubChemE (166484747), PubChemF (166484853), PubChemH (166484986), PubChemI (166484904), and PubChemJ (66604907) exhibit superior binding affinities and favorable hydrogen bonding interactions relative to the standard, suggesting their potential as effective inhibitors against both target proteins. In contrast, compound PubChemG (166484709) demonstrated activity against *Mtb* but lacked favorable interaction with *Msm*

**Figure 6:**
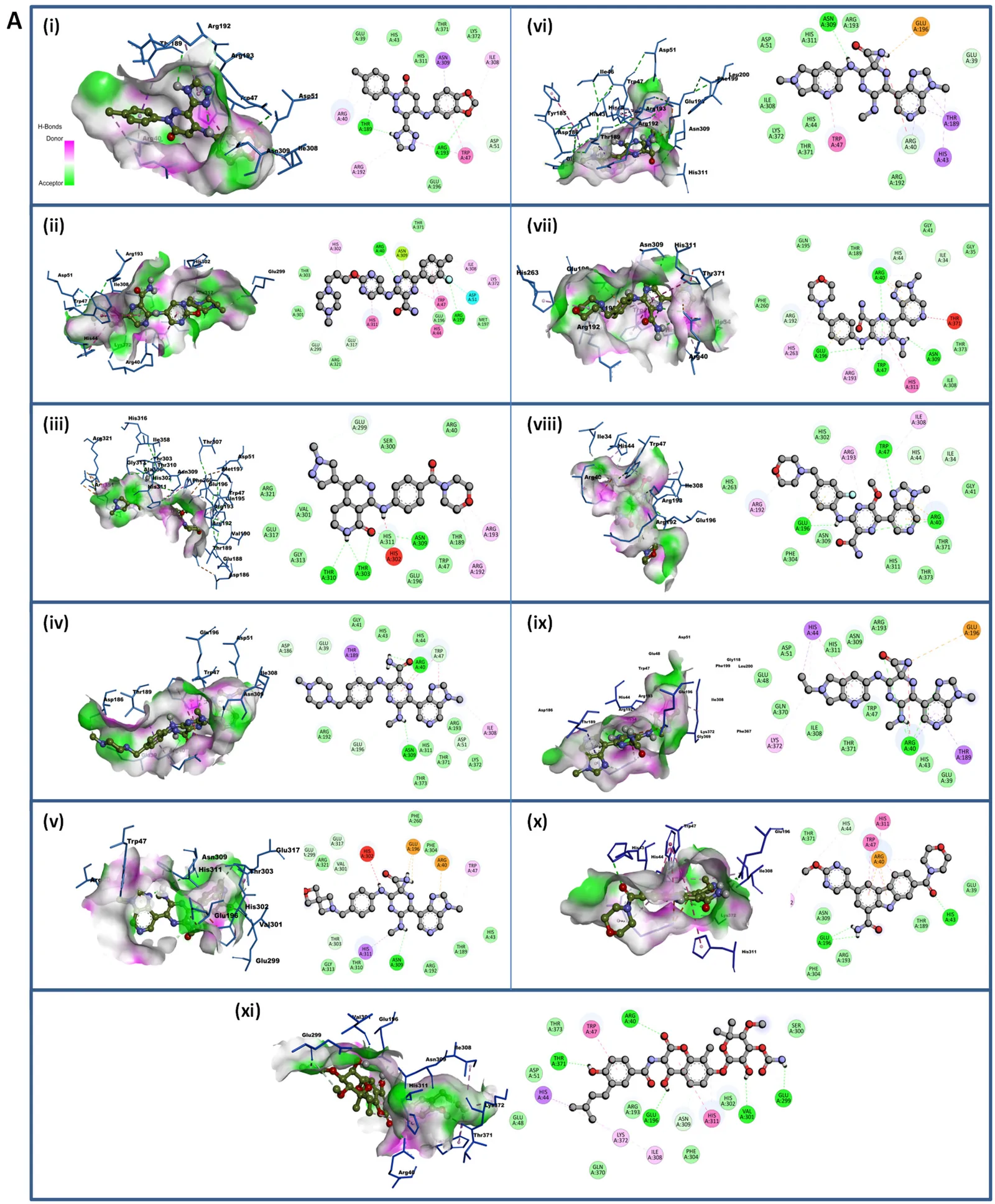

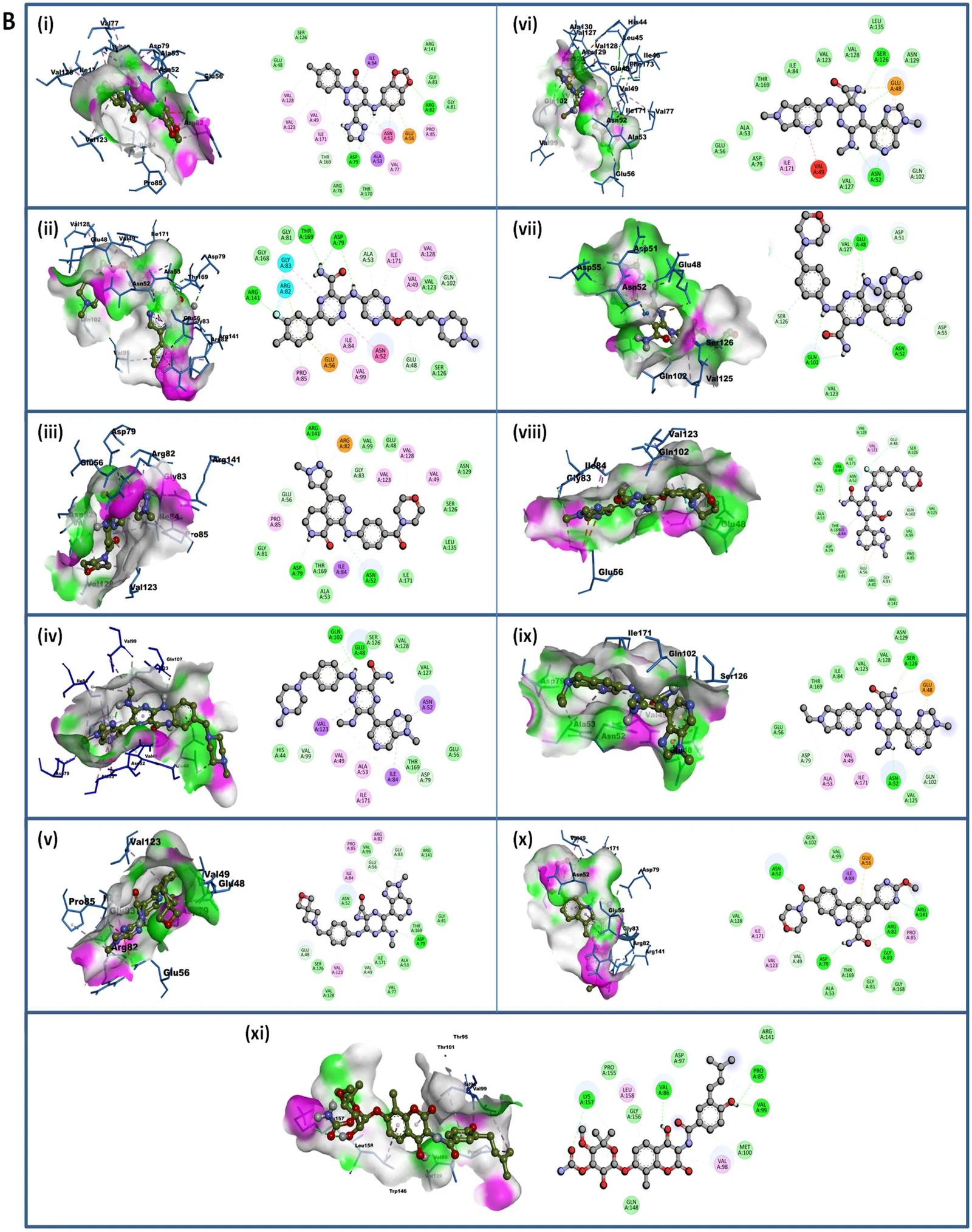
The 2D and 3D molecular docking interactions of the compounds **(A–J) (i)** compound A (ii) compound B (iii) compound C (iv) compound D **(v)** compound E (vi) compound F (vii) compound G (viii) compound H (ix) compound I **(x)** compound J and (xi) novobiocin against the target proteins **(A)** *Mtb*: 3ZKB **(B)** *Msm*: 4B6C.

**Figure 7:**
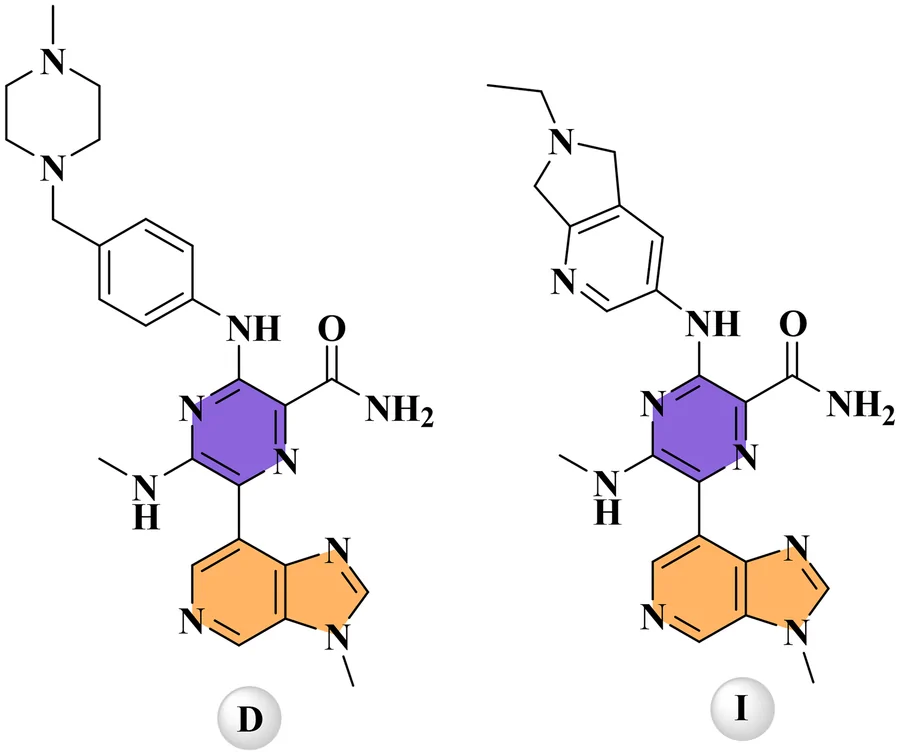
Chemical structures of lead compounds, compound D & I.

**Figure 8:**
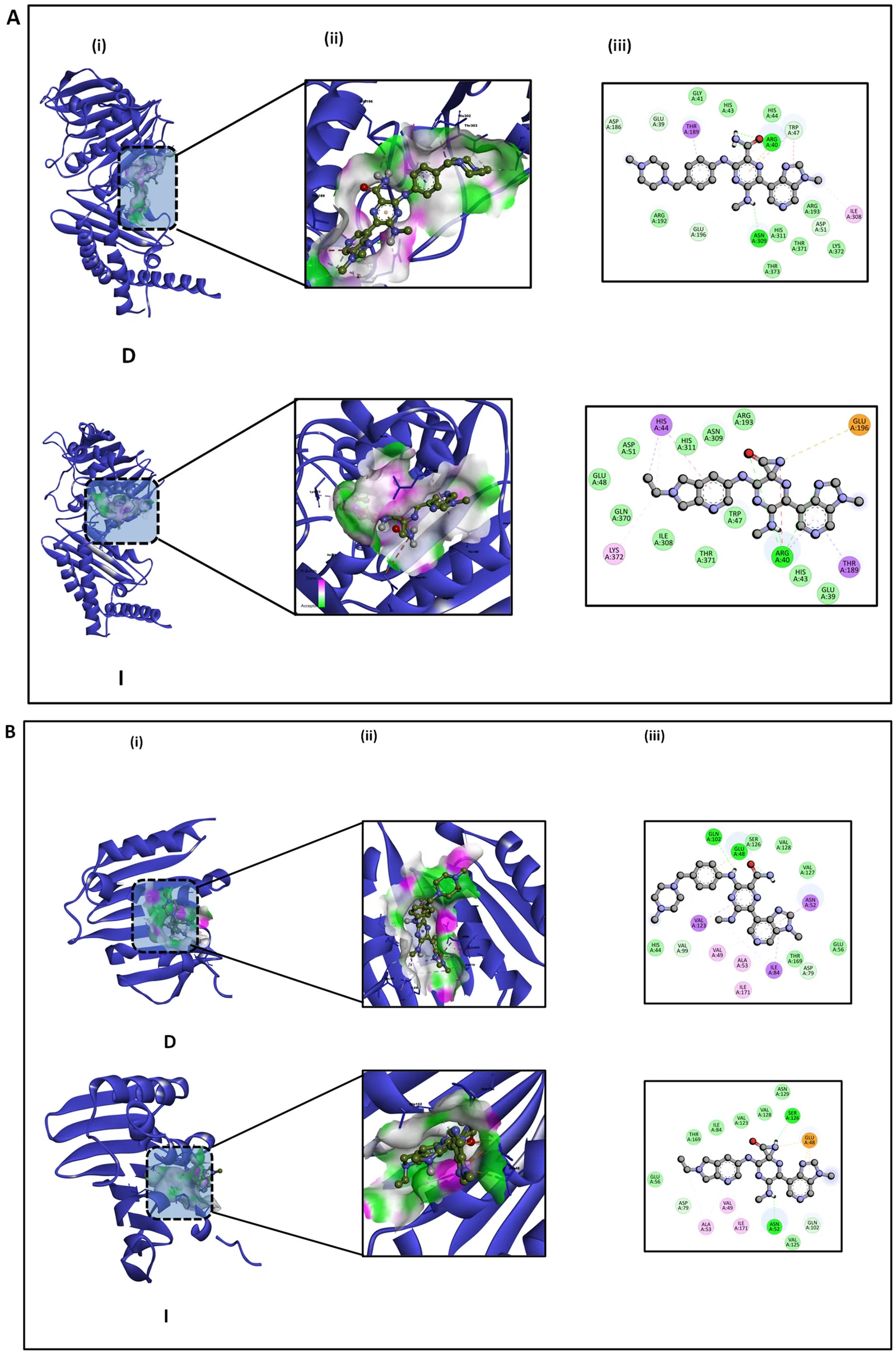
Binding interaction analysis of lead compounds D and I with DNA gyrase B of **(A)** *Mtb* (PDB ID: 3ZKB) and **(B)** *Msm* (PDB ID: 4B6C). **(i)** Overall three-dimensional structure of the protein showing ligand binding within the active site. (ii) Enlarged view of the binding pocket illustrating key ligand–residue interactions. (iii) Two-dimensional interaction diagrams depicting the interactions of the ligands with active-site residues.

Overall, the identified compounds, particularly PubChemA, PubChemD, and PubChemI, demonstrated superior binding affinities and favorable interaction profiles compared to the standard inhibitor, indicating their potential as promising GyrB-targeted anti-mycobacterial candidates for further investigation. Among these, compounds D (166484985) and I (166484904) were identified as the most promising leads due to their consistently high binding affinities and stable interactions with the GyrB subunit of both targets, whereas compound A was excluded from further consideration due to its predicted carcinogenicity. Both compounds exhibited superior docking performance compared to the standard ligand and showed consistent hydrogen-bonding interactions with key active-site residues. Notably, toxicity predictions indicated that both compounds are inactive across all evaluated parameters, suggesting a favorable safety profile. Based on these findings, compounds D and I have been selected as lead molecules for further studies, including molecular dynamics simulations and DFT calculations, to better understand their stability and electronic properties. The chemical structures of the lead compounds D and I are presented in Figure 6, while their detailed binding interaction profiles with DNA gyrase B of *Mtb*(3ZKB) and *Msm*(4B6C) are illustrated in Figures 7, 8.

### 3.6 Molecular dynamics simulation

#### 3.6.1 Simulation studies of *M.tuberculosis* gyrB (3ZKB)

**Figure 9:**
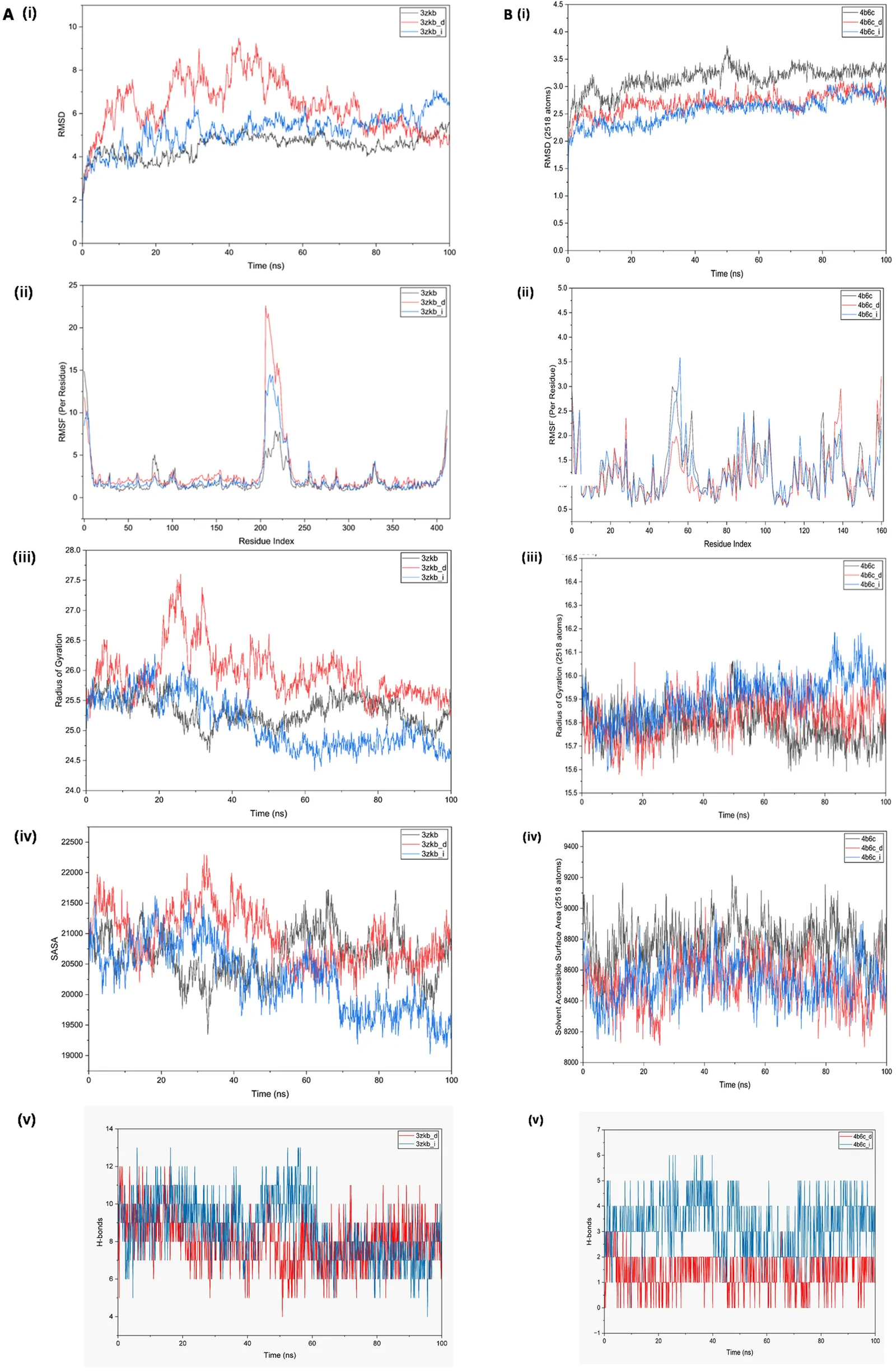
Molecular dynamics simulation analysis of DNA gyrase B–ligand complexes of **(A)** Mtb(PDB ID: 3ZKB) and **(B)** Msm (PDB ID: 4B6C). **(i)** Root mean square deviation (RMSD) plots illustrating the structural stability of the protein–ligand complexes over the simulation time. (ii) Root mean square fluctuation (RMSF) plots representing residue-wise flexibility of the protein backbone. (iii) Radius of gyration (Rg) plots indicating the compactness of the protein structure throughout the trajectory. (iv) Solvent accessible surface area (SASA) plots depicting the variation in solvent-exposed surface area over time. **(v)** Hydrogen bond analysis showing the number and stability of intermolecular hydrogen bonds formed between the ligand and protein during the simulation period.

The integrated analysis of the molecular dynamics trajectories highlights a clear contrast between the behaviour of the Apo 3ZKB protein and its complexes with Compounds D and I, at both global and local scales. The Apo form rapidly equilibrates and maintains an average RMSD of approximately 4.5 Å, serving as a stable structural reference for evaluating ligand-induced effects. In comparison, the complex with Compound D displays a marked loss of stability, whereas the complex with Compound I preserves and even enhances structural integrity, consistent with a stabilising, likely inhibitory binding mode. Global structural stability, assessed by RMSD [Figure 9A-(i)], shows that the Compound D complex undergoes a pronounced destabilisation between 20 and 40 ns, with a spike up to approximately 9.5 Å. This large deviation, coupled with the failure to fully re-equilibrate even after 50 ns, indicates that Compound D drives the protein into expanded, non-native conformations rather than into a well-defined alternative state. In contrast, the compound I complex maintains RMSD values within approximately 5.0–6.0 Å over the 100 ns trajectory, closely tracking the Apo protein. This pattern implies that Compound I is well accommodated in the binding pocket and does not disrupt the global fold; instead, it stabilises a bound ensemble that remains structurally coherent.

Local flexibility analysis (RMSF) [Figure 9A-(ii)] pinpoints a loop spanning residues 200–220, likely the ATP-binding lid or an active-site loop, as the principal dynamic element. In the presence of Compound D, this loop becomes hyper-flexible, with fluctuations exceeding 22 Å, suggesting steric clash, weak anchoring, or an inability of the loop to close properly over the ligand. Such excessive disorder at a key regulatory element is incompatible with a well-ordered functional state. By contrast, Compound I significantly reduces the flexibility of this region to ∼14 Å, representing an ∼8 Å reduction relative to Compound D. This dampening of loop dynamics indicates stabilizing interactions between I and the loop residues that effectively tether the segment, reducing entropic penalties and favouring a more ordered, potentially inactive conformation.

Measures of structural compactness via the Radius of Gyration (Rg) [Figure 9A-(iii)] reinforce these observations. For the Compound D complex, Rg increases sharply above 27.5 Å during the same 20–40 ns window as the RMSD spike, indicating substantial expansion or partial unfolding of the protein domains. This expanded state, together with elevated RMSD and loop hyper-flexibility, points toward a destabilized conformational ensemble. In contrast, the Compound I complex exhibits progressive compaction; within the 50–100 ns interval, Rg stabilizes at ∼24.5 Å, falling below that of the Apo protein. This “tightening” suggests that Compound I promotes a super-compact arrangement, consistent with a locked or inhibitory state in which domain motions are restricted and the protein is held in a conformationally constrained form.

Solvent Accessible Surface Area (SASA) [Figure 9A-(iv)] provides an energetic perspective that aligns with structural metrics. For the Compound D-bound system, SASA exceeds 22,000 Å^2^ during structural expansion, indicating exposure of the hydrophobic core to solvent and an energetically unfavourable, destabilised state. The simultaneous rise in Rg and SASA clearly supports the idea of partial unfolding caused by Compound D binding. In contrast, the Compound I-bound complex shows a gradual decrease in SASA, reaching values below 19,500 Å^2^ by the end of the simulation. This reduction reflects effective shielding of the binding pocket and burial of hydrophobic residues, suggesting that Compound I “zips” the cavity shut and maintains a tightly packed, solvent-excluded environment typical of strong and specific binding. Hydrogen-bond analysis [Figure 9A-(v)] further emphasizes the difference between the two ligand-bound systems. Both complexes form multiple hydrogen bonds during the simulation, confirming that the ligand remains within the binding pocket; however, the strength and duration of these interactions vary notably. The Compound I complex consistently shows higher hydrogen-bond occupancy, usually between around 6 and 12 bonds, with several extended periods of strong interactions throughout the simulation. Notably, in the middle phase (∼40–65 ns), hydrogen bonds remain stable, indicating persistent polar interactions between the ligand and active-site residues. This stable hydrogen-bond network aligns with lower RMSF, reduced SASA, and increased compaction seen in the I-bound system, suggesting that Compound I effectively secures and stabilizes the protein in a compact inhibitory form. Conversely, the Compound D complex forms fewer hydrogen bonds, generally around 5–9, with more fluctuation and periodic drops in occupancy. These inconsistent interaction patterns correlate with higher RMSD, increased loop flexibility, and occasional structural expansion, signaling weaker and less coordinated stabilization. Overall, although both ligands stay bound to the protein, Compound I creates a more enduring and supportive hydrogen-bonding network that greatly enhances the stability of the complex.

Binding free-energy analysis further validates the superior stability of the Compound I complex. Compound I exhibits a more favourable binding free energy (−45.0285 kcal/mol) compared with Compound D (−39.1122 kcal/mol), indicating stronger overall affinity toward the 3ZKB protein. The lower free-energy value of Compound I is fully consistent with its stable RMSD profile, reduced loop flexibility, progressive structural compaction, lower SASA values, and persistent hydrogen-bonding network observed throughout the simulation. These results collectively suggest that Compound I forms a thermodynamically stable and tightly packed protein–ligand complex. In contrast, the comparatively less favourable free energy of Compound D aligns with its destabilising structural behaviour, including increased conformational expansion, elevated solvent exposure, and weaker intermolecular interaction persistence. The free-energy results therefore provide energetic confirmation that Compound I stabilises the protein more effectively and achieves stronger binding than Compound D.Taken together, the integrated RMSD, RMSF, Rg, and SASA, hydrogen-bond, and free-energy analysesresults indicate that Compound D is a destabilising ligand that promotes expanded, disordered, and energetically unfavourable conformations of 3ZKB, particularly in the crucial 200–220 loop region. In contrast, Compound I stabilizes both the global protein scaffold and the local regulatory loop, driving the system toward a compact, solvent-shielded, and dynamically restrained ensemble. The persistent hydrogen-bonding network and more favourable binding free energy associated with Compound I further support its ability to maintain a tightly packed and structurally coherent inhibitory state. This unified view of structure, dynamics, and solvent exposure, intermolecular interactions, and energetic stability strongly supports Compound I as a more promising candidate for further development, consistent with a ligand that stabilizes a functionally restricted-likely inhibitory-conformational state of the protein.

#### 3.6.2 Simulation studies of *Msm* (4B6C)

The RMSD [Figure 9B-(i)] trajectories indicate that the Apo protein explores the largest conformational space, with backbone deviations around 3.0–3.5 Å, consistent with a flexible, unliganded state capable of sampling multiple conformers. In contrast, both ligand-bound systems exhibit lower and more stable RMSD plateaus, demonstrating that ligand binding limits large-scale backbone motion and anchors the protein near its initial structure. Compound I stabilises the protein in an intermediate regime (≈2.5–3.0 Å), which is clearly more ordered than the Apo form yet retains some conformational flexibility. Compound D, however, maintains the lowest and flattest RMSD values (≈2.5–2.7 Å), indicating that it functions as a strong “stiffening” ligand that locks the protein into a conformation very close to the crystal or docked structure. This pattern supports the view that Compound D imposes stronger structural constraints, while I achieve stabilisation without over-rigidifying the fold.

Residue-wise fluctuations [Figure 9B-(ii)] reveal how each ligand shapes local dynamics and help map the likely binding site. The Apo protein shows pronounced flexibility around residues 50–60 and 130–140, behaviours typical of surface loops or functional segments that respond strongly to the absence of a ligand. Upon binding, both Compound D and I drastically dampen the fluctuations at residues 50–60, collapsing the Apo RMSF peak (∼2.0 Å) to much lower values in the complexes. This convergent reduction strongly supports the assignment of residues 50–60 as the primary binding pocket or a key part of the active site, where both ligands engage and locally rigidify the structure. In the 130–140 region, Compound D again suppresses motion substantially relative to Apo, indicating broader allosteric stabilization or reduced loop breathing. By comparison, Compound I leaves this distal loop slightly more mobile than D, suggesting that Compound I either interacts differently with this region or permits a distinct dynamic substate, potentially relevant for functional breathing or allosteric signalling. Overall, the RMSF behaviour confirms that both ligands dampen random thermal motion at the binding site while Compound D exerts a more extensive rigidifying influence.

Compactness analysis through Rg [Figure 9B-(iii)] offers a global structural context for these local effects. The Apo and Compound I-bound systems display similar Rg values (≈15.8–16.1 Å), suggesting that Compound I stabilises the protein without significantly changing its overall size or folding volume. The slight increase in Rg toward the end of the Compound I trajectory indicates modest “breathing” of the protein, consistent with a bound state that maintains some conformational flexibility to accommodate the ligand or enable subtle functional motions. Conversely, the Compound D-bound complex shows consistently lower Rg values, typically below 15.9 Å, emphasising a more densely packed structure. This increased compactness correlates with the reduced RMSD and broader damping of RMSF, portraying D as a ligand that not only stabilises the backbone but also pulls the structure into a tightly folded, less fluctuating state. Such behaviour aligns with a strongly inhibitory or conformationally restrictive binding mode.

SASA [Figure 9B-(iv)] profiles complement the Rg data by describing how ligand binding alters solvent exposure and pocket closure. The Apo protein displays the highest SASA (≈8600–9000 Å^2^), consistent with an open, solvent-exposed conformation typical of a flexible, unoccupied binding site. Both Compound D and I reduce SASA relative to Apo, a hallmark of effective binding in which the ligand buries previously exposed surfaces and promotes partial closure of the protein around the inhibitor. This demonstrates that both compounds occupy the pocket in a well-integrated manner rather than associating superficially at the surface. Compound D generally exhibits the lowest SASA among the systems, in agreement with its reduced Rg and strongly rigidified character; the protein in the D- complex appears more tightly closed and better shielded from solvent. Compound I, while still clearly reducing SASA compared to Apo, maintains slightly higher exposure than D, consistent with a bound state that is less compressed and retains a modestly more open conformation.

Hydrogen bond analysis further supports the differential stabilisation patterns observed for the two ligand-bound systems [Figures 9B-(v)]. The Compound I complex maintains a consistently higher number of hydrogen bonds throughout the simulation, generally fluctuating between 3 and 6 interactions and occasionally reaching higher values, indicating persistent and stable intermolecular contacts within the binding pocket. In contrast, the Compound D complex exhibits fewer hydrogen bonds, typically around 1–2, with more frequent interruptions and transient losses of interaction. The sustained hydrogen-bonding network in the Compound I system suggests stronger local interaction stability and improved binding persistence over the trajectory. Meanwhile, the comparatively reduced hydrogen-bond occupancy in the Compound D system implies that its stabilising effect may arise more from hydrophobic packing and conformational restriction than from extensive polar interactions. Together, the hydrogen-bond profiles indicate that although both ligands stabilise the protein structure, Compound I achieves this through a more persistent interaction network, whereas Compound D promotes rigidity primarily through compact structural locking.

Binding free energy calculations provide further insight into the energetic basis of these findings. Compound D shows a significantly more favourable binding free energy (−55.0526 kcal/mol) compared to Compound I (−44.3320 kcal/mol), indicating a stronger overall affinity for the *Msm* gyr B protein. The lower free energy of Compound D aligns with its reduced RMSD, RMSF, Rg, and SASA values, all suggesting the formation of a highly compact and conformationally constrained complex. Although Compound D forms fewer hydrogen bonds than Compound I, it demonstrates superior energetic stability, implying that non-polar interactions, van der Waals forces, and efficient packing within the binding pocket play key roles in its binding strength. Conversely, Compound I relies more on persistent hydrogen bonds to stabilize the complex, resulting in a more dynamic yet still favorable bound state. These results emphasize that hydrogen-bond count alone does not determine binding affinity; instead, the overall interplay of polar and hydrophobic interactions governs the energetic stability of the protein–ligand complex.

Taken together, the RMSD, RMSF, Rg, SASA and H-bond analyses provide a clear picture: both Compounds D and I are plausible stabilisers of the *Smeg* protein, engaging the binding pocket around residues 50–60 and reducing overall flexibility compared to the Apo state. Compound D acts as the more robust rigidifier, maintaining a compact, low-RMSD, low-Rg, and low-SASA conformation that likely represents a tightly inhibited or structurally locked state. In contrast, Compound I stabilises the protein while allowing a slightly higher degree of global breathing and regional flexibility, particularly around residues 130–140 and simultaneously maintains a more persistent hydrogen-bonding network with the protein. This suggests that Compound I supports a related but dynamically distinct conformational substate driven by stable polar interactions. From a design standpoint, Compound D seems optimal for achieving maximum structural locking, whereas Compound I could be useful in situations where some residual conformational flexibility is beneficial, such as fine-tuning allosteric regulation or avoiding excessive restriction of functionally important motions.

### 3.7 DFT results

#### 3.7.1 Frontier molecular orbitals (FMO)studies

**Figure 10:**
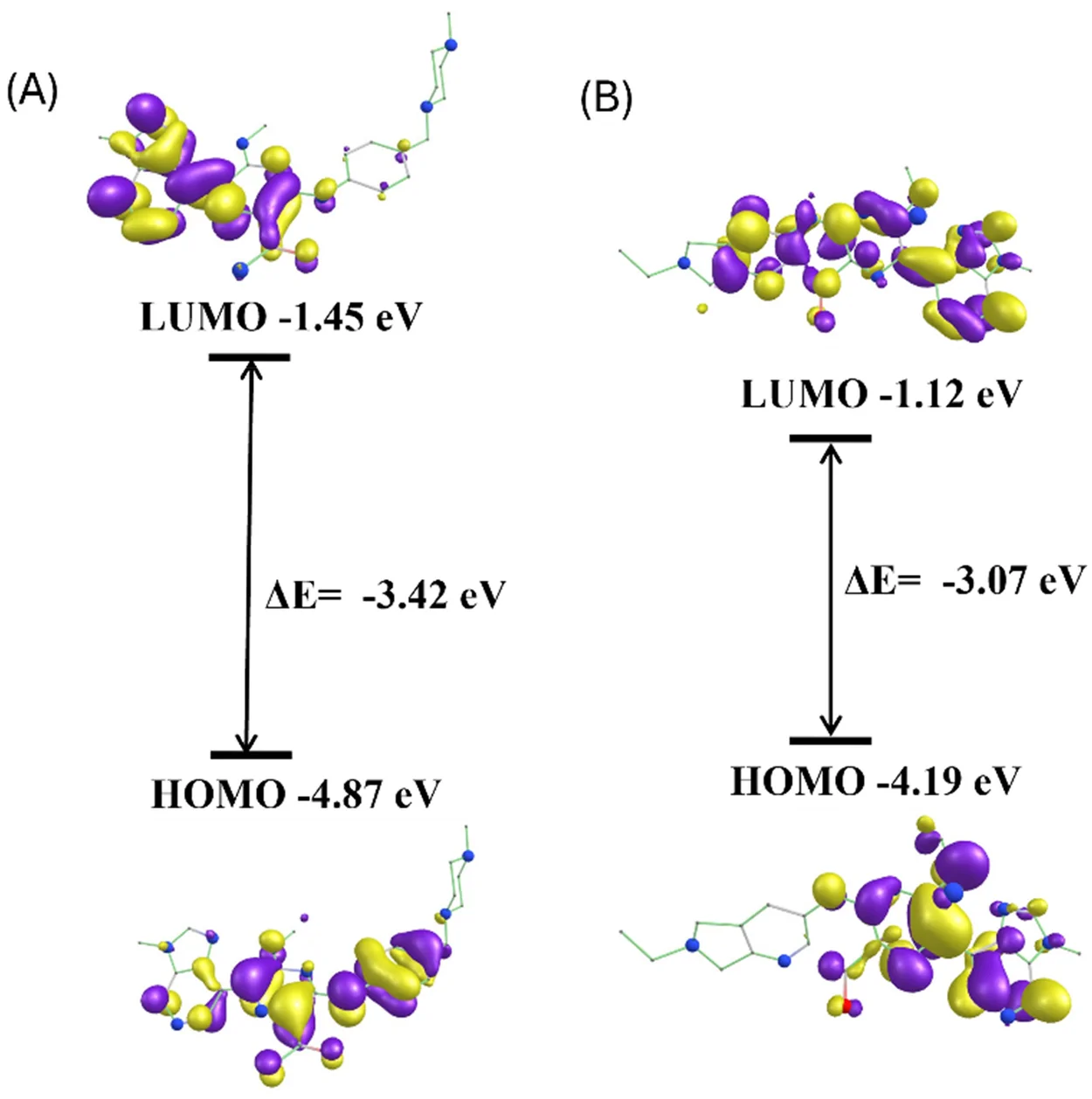
DFT-calculated HOMO–LUMO orbitals and corresponding energy gaps of **(A)** compound D and **(B)** compound I at the B3LYP/6–31G** level of theory. Orbital isovalue=0.03; purple and yellow regions represent positive and negative orbital phases, respectively. Hydrogen atoms were omitted for clarity.

The DFT-derived electronic descriptors were analyzed to better understand the interaction potential and electronic stability of the selected compounds within the ATPase binding pocket of DNA Gyrase B. The HOMO and LUMO distributions revealed potential electron donor and acceptor regions that may participate in intermolecular interactions with active-site residues. Moreover, the HOMO–LUMO energy gap provides important insights into molecular reactivity and global electronic descriptors, including chemical hardness, chemical potential, and electrophilicity. The HOMO and LUMO energy levels, together with their corresponding energy gaps, are presented in Figure 10. The negative HOMO and LUMO energy values observed for both compounds further support their electronic stability (49).

Among the investigated compounds, Compound I displayed HOMO and LUMO energies of −4.19 eV and −1.12 eV, respectively, with an energy gap of 3.07 eV, whereas Compound D exhibited HOMO and LUMO energies of −4.87 eV and −1.45 eV, respectively, with a larger HOMO–LUMO energy gap of 3.42 eV. The larger HOMO–LUMO energy gap and higher electrophilicity index of Compound D indicate greater electronic stability and an enhanced ability to participate in stabilizing charge-transfer interactions within the ATPase pocket.

Electronegativity (*χ*) quantifies a ligand's ability to attract electrons, thereby influencing its interactions at binding sites and within the surrounding protein environment. Several chemical reactivity parameters, including electronegativity (*χ*), chemical potential (µ), global electrophilicity index (*ω*), global hardness (*η*), and global softness (S), were calculated using the chemical equations described below, and the results are summarized in Table 5. Compound D showed higher electronegativity (*χ* = 3.16) and global hardness (*η* = 1.71), indicating greater resistance to charge transfer and increased stability. It also had a higher electrophilicity index (*ω* = 5.83) than Compound I (*ω* = 4.58), implying a stronger tendency to accept electrons in intermolecular interactions. Collectively, these findings indicate that Compound D possesses more favorable DFT-derived electronic characteristics, which may contribute to its improved interaction stability and binding persistence within the DNA Gyrase B ATPase active site.

$$\mu =\;\frac{\left(E_{HOMO}+E_{LUMO}\right)}{2}$$

$$\chi =\;-\frac{\left(E_{HOMO}+E_{LUMO}\right)}{2}$$

$$\eta =\;\frac{\left(E_{HOMO}-E_{LUMO}\right)}{2}$$

$$\sigma =\;\frac{1}{\eta }$$

$$S=\;\frac{1}{2\eta }$$

$$\omega =\;\frac{\mu 2}{\eta }$$

**Table 5:** Quantum chemical parameters for the investigated compounds using B3LYP/6–31G** level of theory.

S. No.	Properties	Compound D	Compound I
1.	μ	−3.16	−2.65
2.	χ	3.16	2.65
3.	η	1.71	1.53
4.	**σ**	0.58	0.65
5.	s	0.29	0.32
6.	*ω*	5.83	4.58

Interestingly, although Compound D forms fewer hydrogen bonds in *Msm* GyrB, it shows greater overall stability than Compound I. This suggests that Compound D's enhanced electronic stability and electrophilicity may strengthen hydrophobic and van der Waals interactions, offsetting the fewer hydrogen-bonding interactions and leading to better complex stability. Conversely, in the *Mtb* GyrB system, Compound I form more hydrogen bonds and has higher binding stability than Compound D, indicating that hydrogen-bonding stabilization is more significant in the *Mtb* ATPase pocket. Overall, these results demonstrate that ligand stability in the gyrase B active site depends not only on hydrogen-bond count but also on the interplay of electronic characteristics, van der Waals forces, and the specific microenvironment of the target protein. The DFT findings align with the docking and molecular dynamics results, supporting the distinct interaction patterns of Compounds D and I in *Msm* and *Mtb* GyrB systems.

## 4 Conclusion

The N-terminal ATPase domain of DNA Gyrase B in *Mycobacterium tuberculosis* (*Mtb*) and *Mycobacterium smegmatis* (*Msm*) is highly conserved in sequence, structure, and catalytic function, exhibiting ∼85%–87% sequence identity and nearly identical ATP-binding pocket geometry (RMSD < 1 Å). Essential catalytic residues involved in ATP binding and hydrolysis, including Glu48, Asn52, Asp73, Asp118, Arg141, Arg180, and Lys372, are strictly conserved, supporting preservation of Mg–ATP coordination and catalytic activity (50). Most sequence variations are conservative and surface-exposed; however, localized differences within the ATP-lid (100–130 residues) and transducer/C-loop region (200–245 residues) induce subtle conformational changes that influence ATPase dynamics and inhibitor accommodation. A notable variation at residue 208 (Ser in *Mtb* and Thr in *Msm*) within the inhibitor-binding pocket may further contribute to species-dependent interaction behavior.

Despite the strong conservation of the catalytic and drug-binding cores, localized structural variations appear to modulate inhibitor affinity and ATPase regulation, explaining the distinct binding propensities of Compounds D and I toward *Mycobacterium tuberculosis* and *Mycobacterium smegmatis* GyrB. Compound D demonstrated enhanced electronic stability and stronger van der Waals-driven stabilization in *Msm* despite fewer hydrogen-bonding interactions, whereas Compound I exhibited greater hydrogen-bonding interactions and superior stability in the *Mycobacterium tuberculosis* ATPase pocket. These findings indicate that ligand stabilization is governed not only by hydrogen bonding but also by electronic properties, hydrophobic contacts, and local pocket dynamics.

Compounds D and I, containing pyrazine/aminopyrimidine-linked fused heterocyclic scaffolds, exhibited pharmacophoric features comparable to reported ATPase-targeting GyrB inhibitors, including Novobiocin and aminopyrazinamide derivatives (28, 51). Their heteroatom-rich cores and aromatic frameworks support favorable hydrogen-bonding and hydrophobic interactions within the ATPase cavity while providing structurally distinct scaffolds that may overcome limitations associated with classical aminocoumarins.

Similar to recent translational computational studies targeting mycobacterial proteins, the combined evaluation of binding affinity, conformational stability, and electronic properties improved the predictive reliability of the identified lead compounds and provided a robust framework for future experimental validation (7, 52). Unlike previous studies primarily focused on GyrA-targeting fluoroquinolones, the present study explored ATP-competitive inhibition of the conserved GyrB ATPase domain in both *Mycobacterium tuberculosis* and *Mycobacterium smegmatis*, providing a comparative computational framework for anti-tubercular lead discovery.

Overall, the integrated computational workflow, which included pharmacophore modeling, molecular docking, ADMET prediction, molecular dynamics simulations, and DFT analyses, identified Compounds D and I as promising ATP-competitive GyrB inhibitors with distinct species-specific interaction profiles. The combined structural, dynamic, and electronic analyses strongly support their potential as anti-tubercular lead compounds and provide a robust foundation for future experimental validation through *in vitro*, *in vivo*, and pharmacokinetic studies.

## 5 Future prospect

The present in-silico study provides a strong computational foundation for the identification of novel DNA Gyrase B ATPase inhibitors targeting *Mycobacterium tuberculosis* (*Mtb*) and *Mycobacterium smegmatis* (*Msm*). Despite the robust predictive insights obtained through the integrated computational workflow, the findings remain theoretical and require comprehensive experimental validation to establish their therapeutic relevance. Future investigations should initially focus on validating the ATPase inhibitory potential of Compounds D and I through *in vitro* enzymatic inhibition assays, followed by antimycobacterial susceptibility testing against both drug-sensitive and drug-resistant *Mtb* strains. In addition, cytotoxicity evaluation and selectivity profiling against human homologous targets will be essential for assessing safety and minimizing potential off-target effects.

Further lead optimization through systematic structure–activity relationship (SAR) studies and rational chemical modifications may improve potency, selectivity, and pharmacokinetic behavior. Advanced computational approaches, including longer-timescale molecular dynamics simulations, free-energy perturbation (FEP) analyses, and fragment-based drug design strategies, could provide a more accurate estimation of binding stability and facilitate the identification of cryptic or allosteric regulatory sites within the GyrB ATPase domain. Moreover, multi-target profiling against other essential mycobacterial enzymes, such as DNA Gyrase A, topoisomerase I, and cell-wall biosynthesis proteins, may help evaluate broader antimycobacterial potential and possible synergistic mechanisms.

To further strengthen translational applicability, advanced toxicity prediction models, metabolic stability studies, and *in vivo* pharmacokinetic and pharmacodynamic investigations will be necessary to validate drug-likeness beyond theoretical ADMET predictions. From a broader drug-discovery perspective, integrating artificial-intelligence-driven molecular generation, machine-learning-assisted virtual screening, and large-scale exploration of chemical libraries may significantly accelerate the development of next-generation ATPase-targeting GyrB inhibitors. Combining computational modeling with biochemical, microbiological, and preclinical validation may aid in developing selective, resistance-resistant anti-tubercular drugs targeting the conserved ATPase pocket of DNA Gyrase B.

## Data Availability

The original contributions presented in the study are included in the article/Supplementary Material, further inquiries can be directed to the corresponding author/s.
