# Supplementary material for: Identification of novel DNA gyrase B inhibitors in Mycobacterium species using integrated computational approaches
Source: Front Tuberc. 2026 Jun 18;4:1854329. doi: 10.3389/ftubr.2026.1854329 (PMC13491435; doi:10.3389/ftubr.2026.1854329)
Supplement: Supplementary file 1 [file Datasheet1.docx]

| S.No | Compounds | *M. tuberculosis* (3ZKB) | | *M. smegmatis* (4B6C) | |
| --- | --- | --- | --- | --- | --- |
|  |  | **Score**  **(kcal/mol)** | **Hydrogen/**  **Remarkable bonds** | **Score**  **(kcal/mol)** | **Hydrogen/**  **Remarkable bonds** |
| 1 | D | -7.48 | His 43, Arg 40**(H bond),** Asn 309 , Arg 193, Glu 39, Asp 186 | -5.01 | Gln 102, Asn 52**(H bond),** Val 54,Ser 126, Ala 53,Glu 48, Val 123, Asp 79, Val 127 |
| 2 | I | -6.80 | Thr 189, Trp 47, Arg 192**(H bond),** Lys 372, His 44, Arg 40,Glu 196,His 311, Asn 309 | -5.25 | Arg 141, Gly 83 **(H bond),** Ile 84, Ile 171, Asp 79, Ala 53, Val 127,Asn 52(Alkyl), Asp 79,Thr 169, Glu 56 |

**TITLE: Identification of Novel DNA Gyrase B Inhibitors in Mycobacterium species Using Integrated Computational Approaches**

**Jagritee Yadav^a^, Lalhmangaihzuali^b^, Harish Shukla^b^, Anshuman Chandra^c^, Sudhir Kumar Singh^d^*, Meenakshi Singh^a^*.**

^a^Department of Medicinal Chemistry, Faculty of Ayurveda, Institute of Medical Sciences, Banaras Hindu University, Varanasi 221005, India

^b^Department of Biochemistry, North Eastern Hill University, Shillong, Meghalaya 793022, India

^c^School of Physical Sciences, Jawaharlal Nehru University, New Delhi 110067, India

^d^Department of Microbiology, Virus Research and Diagnostic Laboratory (VRDL), Institute of Medical Sciences, Banaras Hindu University, Varanasi 221005, India

Corresponding author*:[sudhirsingh.mku@gmail.com](mailto:sudhirsingh.mku@gmail.com); [meenakshisingh@bhu.ac.in](mailto:meenakshisingh@bhu.ac.in)

**Electronic Supplementry Material (ESM)**

**Table S-1:** Molecular docking results of lead compound(D & I) against the dimeric GyrB subunit of *Mtb*  (PDB ID: 3ZKB) and *Msm* (PDB ID: 4B6C)

**Fig S-2.** The 2D and 3D molecular docking interactions of the (i) Compound D & (ii) Compound I against the dimeric target proteins (A) *Mtb*: 3ZKB (B) *Msm*: 4B6C
